# Caring helps: Trait empathy is related to better coping strategies and differs in the poor versus the rich

**DOI:** 10.1371/journal.pone.0213142

**Published:** 2019-03-27

**Authors:** Rui Sun, Laura Vuillier, Bryant P. H. Hui, Aleksandr Kogan

**Affiliations:** 1 Department of Psychology, University of Amsterdam, Amsterdam, Netherlands; 2 Department of Psychology, Bournemouth University, Poole, United Kingdom; 3 Department of Sociology, University of Hong Kong, Hong Kong SAR, China; 4 Department of Psychology, University of Cambridge, Cambridge, United Kingdom; Utrecht University, NETHERLANDS

## Abstract

Coping has been extensively studied in health psychology; however, factors influencing the usage of different coping strategies have received limited attention. In five studies (*N* = 3702), we explored the relationship between trait empathy and coping strategies, and how subjective socioeconomic status (SES) moderates this relationship. In Studies 1–4, we found that people with higher level of empathic concern use more adaptive coping strategies, seek more social support, and use fewer maladaptive coping strategies. Moreover, higher trait empathy related to more adaptive coping strategies among the poor, and fewer maladaptive coping strategies among the rich. In Study 5, we tested the potential biological basis of the relationship between trait empathy and coping by examining the effect of the oxytocin receptor gene (OXTR) rs53576 polymorphism on coping. We found that individuals with the GG phenotype—who in previous research have been found to be more empathic—were more likely to seek social support than AG or AA individuals. Furthermore, in line with findings in Studies 1–4, amongst people with low SES, individuals with GG genotype used more adaptive coping strategies than AG or AA individuals. Our results highlight the selective role trait empathy plays in influencing coping strategy deployment, depending on the SES of individuals.

## Introduction

Humans encounter various obstacles and difficulties during the course of their lives, ranging from daily frustrations to significant life events. How one copes with stress influences health outcomes and well-being (see [1] for a review). Canonically defined as thoughts and behaviours people use to manage the interplay of external and internal demands in stressful situations [2], coping strategies can be summarised into adaptive and maladaptive ones based on whether they help reduce the negative effect of stress, leave it unchanged, or make it worse [3]. Given the near ubiquity of stress across life domains [4], understanding the factors that promote healthier usage of coping strategies has deep implications for individuals and policy makers who are interested in boosting mental and physical health.

Within psychology, a number of studies have suggested a myriad of different factors that are likely to influence coping strategies, such as sociodemographic factors of education and income [5], personality dispositions of self-confidence [6], contextual factors of negative life events [7–9], and family support [10]. One factor that has yet to receive much attention, however, is empathy: The ability to feel and understand others’ thoughts and emotions [11]. In the present work, we build upon theory in the study of empathy to propose that it can act as a promoter of adaptive coping and an inhibitor of maladaptive coping. Further, our model suggests that socioeconomic status (SES) plays a key role in moderating this relationship because individuals with different SES would benefit differently from trait empathy. We also explored whether the potential biological antecedent of empathy (oxytocin receptor gene rs53576 polymorphism) also plays a role in coping strategies. If so, it would provide additional support that empathy as a trait relates to coping strategies.

We were guided by two main hypotheses: (1) Empathy is positively related to healthier coping strategies such as using more adaptive coping strategies and seeking social support, and/or using fewer maladaptive coping strategies; (2) this relationship is different among lower and higher SES individuals—we tested competing hypotheses about the directionality of this effect. To test these two hypotheses, we conducted a number of correlational studies using both psychological and genetics methods.

### A brief review on coping

Coping has been the subject of extensive investigations in psychological research over the past 40 years [12]. What coping strategies to choose, and whether the coping is effective, depend on a person’s traits (such as personality) and social context (e.g., marital satisfaction) [13]. It is important to note that coping processes are not inherently good or bad, rather, highly contextual-dependent and needed to be evaluated in a specific stressful context [2]. Indeed, the same coping strategy can be adaptive for some people in certain situations but maladaptive for others in other situations. For instance, a child engaging in withdrawal and submissive behaviours in the context of a hostile parenting environment may be adaptive to avoid abuse, but such coping behaviour could be highly maladaptive when moving into adult life where one has to manage different life tasks. Folkman and Lazarus [14–16], for example, have repeatedly emphasised that coping should be thought of as a dynamic process that shifts in nature from stage to stage of a stressful situation.

Although the specifics of the coping responses naturally vary across individuals and contexts, researchers have attempted to group similar types of responses into categories of coping strategies. An influential distinction was proposed by Lazarus and Folkman [2] to contrast problem-focused with emotion-focused response strategies. While the former aim to modify the relationship between the environment and the person through dealing directly with the source of the stress, the latter attempt to regulate emotional distress by altering one’s own response to the stressor. Even though problem-focused and emotion-focused coping can occur together in the same coping context, problem-focused coping is generally more likely in situations where people believe that something constructive can be done about the stressor, while emotion-focused coping is more likely when people believe that the situation is one that must be endured [14,17].

What constitutes the most appropriate high-order structure of coping strategies is still being debated [18,19] and some researchers have suggested to group the coping strategies more flexibly. For example, rather than prescribing a rigid structure of the coping strategies assessed by the questionnaire Brief COPE, Carver [20] recommended that researchers use the Brief COPE flexibly and creatively, such as by suggesting the possibility of only selecting a subset of the subscales. Researchers have found it is often sensible to perform exploratory analysis to determine empirically how the data from their sample is to be analysed [21,22]. In the current study, we adopted this approach when assessing our participants’ coping styles.

### Trait empathy and coping strategies

Empathy refers to the capacity to understand and respond to another person’s thoughts and emotions [23]. Even though there exist various definitions of empathy, it is broadly agreed that empathy is not a single ability but a complex socio-emotional competency that encompasses different interacting components [24,25]; it has at least two components: An automatic affective response, which often entails sharing another’s emotions; and a cognitive capacity to take the perspective of others [26,27]. Both may play a role in the processes involved in coping: empathy has long been considered a contributor to positive social interactions, such as developing affective bonds and understanding [28], and promoting caring actions between people [29].

Empathy is naturally involved in some coping processes especially when coping requires one to engage others in helpful interactions. Perceived empathic self-efficacy, one’s perceived abilities to experience empathy, has been found to positively correlate with adaptive coping strategies (such as active coping) and seeking social support (both emotional and instrumental support), as well as to negatively correlate with maladaptive coping strategy (such as behavioural disengagement) [30]. Extant research has also found that empathy is related to better relationship-focused coping [31,32]. For instance, empathic responding may represent an adaptive way of coping with couples’ interpersonal relationship problems [33]. Besides the specific relationship-focused coping that has predominantly been investigated, there is some indirect evidence that empathy is generally related to coping with stress. For instance, high-empathic individuals are more likely to help others and receive in return stronger social support, which was found to be helpful in coping with stress [34]. Empathy is also a key factor in social bonding, such as taking care of offspring and affiliating with social groups, which creates and maintains social networks, builds social resources, and in turn facilitates the process of coping [35]. While various lines of research show that empathy helps individuals deal with stress, to the best of our knowledge, no study has directly examined how empathy influences the choice of coping strategies. Moreover, no study has explored whether the affective component and cognitive component of empathy influence coping strategies differently. We therefore tested how empathic concern (the affective component) and perspective taking (the cognitive component) influence the use of coping strategies, separately.

We hypothesise that empathy should promote better coping, and we suggest that it could manifest itself in two ways. First by empathy promoting more adaptive coping strategies and/or, second by empathy reducing maladaptive coping strategies. There is little empirical basis to generate a specific prediction about whether empathy will impact one or both clusters of coping strategies; thus, we made no directional prediction about the specific manifestation of the empathy’s positive impact on coping—but aimed to examine adaptive and maladaptive coping strategies separately to pinpoint the effect. We also made no differential prediction in how the affective component and cognitive component of empathy influence coping strategies.

### The moderating effect of SES on trait empathy and coping strategies

Empathy is generally regarded as a positive characteristic; however, we reasoned that the role trait empathy plays in coping may in fact be much complex, varying across people. One possible moderator of the relationship between empathy and coping is socioeconomic status (SES). Defined by how individuals identify themselves with a certain economic group and a certain social class [36], SES can be measured both objectively and subjectively. Objective SES, measured by one’s income, occupation and education level, and subjective SES, measured by perceptions of others’ respect and admiration, usually correlate with each other [37,38] Nevertheless, literature suggests that compared to objective SES, subjective SES has more influence on health, stress-coping and well-being[39].

SES has been found to have great influence on health outcomes and mood-related vulnerabilities [40]. For instance, people of higher SES live longer, enjoy better health, and suffer less from chronic diseases compared to those of lower SES [41]. One would naturally suppose that individuals with high and low SES backgrounds use different coping strategies, as the amount and nature of the stress they meet is different. For example, higher SES individuals face less severe stressors, have larger social networks, and have better access to better health care and support services [42,43]. In addition, better educated individuals usually grow up in more socially supported schools and experience lower levels of bullying in life [44]. Existing work points to an association between SES and reliance on certain coping strategies. One line of research has shown that higher SES individuals are more likely to use more adaptive forms of coping involving flexibility, logical choice, and an adherence to consensual reality, while being less likely to rely on defensive strategies involving rigidity and irrationality [45]. Pearlin and Schooler also showed that better educated and more affluent people were less inclined to use selective ignoring in dealing with marital and occupational problems [5], while Billings and Moos found that better educated respondents were more likely to rely on problem-focused coping strategies and less likely to use avoidance coping [46].

The literature lends itself to two competing hypotheses, which we name the *poor-protection* and the *rich-protection* hypotheses. In the *poor-protection* hypothesis, we suggest that the benefit of empathy on coping is stronger for lower SES individuals than higher SES individuals. This hypothesis derives from the work looking into the negative link between SES and empathy. Lower SES individuals are more compassionate [47], are more accurate in judging others’ emotions[48], and display stronger empathic neural responses [49]. Findings also indicate that lower SES individuals orient themselves to the welfare of others as a mean to adapt to their more hostile environments [50]. Because lower SES individuals experience less personal control and tend to depend on others to achieve desired outcomes [51,52], we suggest that being empathic toward others may be more beneficial for them to cope with stress [53]. Thus, from a functional perspective, the effects of empathy should be stronger for poorer individuals.

In contrast, the *rich-protection* hypothesis suggests that empathy may have a stronger influence in richer rather than poorer individuals. Higher SES individuals have greater control over resources, show reduced dependence on others, and an increased ability to behave freely and independently of others’ wishes and aspirations [54,55]. On a distinct but related concept, Cote and colleagues found that social power played a role in the relationship between empathy and prosocial traits [56]. In three studies, they found that prosocial orientation was more strongly associated with empathy among people who were dispositionally powerful, people who were experimentally induced to feel powerful, and employees in high positions. This suggests that the powerful individuals may be able to better extract the natural benefits of a trait—such as empathy—since they are freer and have less constraints to do so. Similarly, in the rich-protection mechanism, we hypothesise that if empathy in general boosts healthier coping strategies, the rich should see an especially strong display of this effect.

### Oxytocin as the potential biological antecedent of empathy

In addition to our investigation of empathy from a behavioural perspective, we aimed to understand whether the potential biological antecedent of empathy would also have an influence on coping strategies. If that was the case, it would provide extra support that empathy as a trait relates to coping strategies. Particularly, we suggest that the oxytocin receptor gene (OXTR) rs53576 polymorphism might be the antecedent of trait empathy and may relate to coping strategies. Oxytocin is a hypothalamic neuropeptide that is thought to be related to prosociality [57] trust [58], empathy [59], and empathic accuracy [60]. In humans, a single-nucleotide polymorphism (SNP) of an adenine (A) or guanine (G) within intron 3 of the OXTR gene (rs53576) has been associated with differences on empathy and related constructs. For instance, individuals with two G alleles are better at understanding people’s emotions [61], show more compassionate displays toward one’s romantic partner [62], have higher trait empathy and empathic accuracy [63], and engage more in charitable activities [64]. Some meta-analysis and population studies revealed that OXTR rs53576 is indeed associated with empathy and that individuals with the GG genotype of rs53576 show better empathic ability [65,66]. OXTR rs53576 is also related to stress regulation. For example, GG genotype individuals display lower physiological and dispositional stress reactivity than AA/AG individuals [63], women with the GG genotype of rs53576 feel more positive affect after a stressor compared to their AG or AA counterparts [67], and GG genotype individuals display significantly higher levels of sympathetic reactivity to psychological stress, lower awakening cortisol levels, and less variation in salivary cortisol across the day as compared to A carrier individuals [68]. It is important to note however that not all oxytocin receptor gene polymorphisms are related to coping: for example OXTR rs2254298 and rs2268498 do not moderate the relationship between childhood maltreatment and adult depression and anxiety[69], therefore in the current project, we only looked at the most widely studied rs53576 polymorphism. Another point to note is that there is some evidence that psychosocial stress exposure dynamically regulates OXTR, and that epigenetic modification of genes involved in oxytocin signaling might be involved in the mechanisms mediating the long-term influence of early adverse experiences on socio-behavioural outcomes [70]. However, the current study only focuses on the static OXTR influence on coping.

Collectively, the above studies outline the case for oxytocin in general—and the rs53576 OXTR SNP in particular—as being a potential biological antecedent of individual differences in empathy, and how they might be related to coping with everyday stress. However no study, to the best of our knowledge, has tested this hypothesis yet. In line with previous studies, we reasoned that individuals homozygous for the G allele of rs53576 should have higher empathy, and thus (1) use healthier coping strategies, especially in using social support (in line with previous research), and (2) show a moderation of this effect by SES following one of our two competing hypotheses outlined above.

### The present research

The present research tests two core hypotheses across five studies: (1) that empathy is positively related to healthy coping strategies (more adaptive coping strategies, including seeking social support, and/or fewer maladaptive coping strategies), and (2) that SES moderates the relationship between empathy and coping—following either of our two competing hypotheses, poor-protection vs. rich-protection.

In Study 1, we first aimed to identify the structure of coping strategies using exploratory factor analysis. We also tested our hypotheses by looking at the main effect of empathy on coping, and how subjective SES interacts with empathy to influence coping. In Study 2, we validated the structure of coping strategies using confirmative factor analysis, and we aimed to replicate the results from Study 1 in another sample. For both Studies 1 and 2, we recruited our participants on MTurk; however, given the possible problem of a biased participant sample on MTurk [71,72], we recruited participants on two other survey panels for Studies 3 (through Tellwut) and 4 (through Cint). Procedures were otherwise identical in these four studies. Finally, in Study 5, we examined the effect of the genetic variations of OXTR rs53576 in a sample of British Caucasians, recruited from the Cambridge BioResource panel. In this last study, we aimed to determine the potential biological bases of the moderating role of SES on the relationship between empathy and coping.

## Study 1

### Method

#### Participants

Four hundred participants from the United States were recruited via Amazon MTurk for the study. After removing participants who had duplicated entries and those who had incomplete cases, 339 participants (Male = 146, *M*_age_ (*SD*) = 34.18 (8.98)) were included in the analysis.

#### Measures

All the studies received ethical approval from the University of Cambridge Research Ethics Committee. Participants gave written consent to take part in the studies.

**BRIEF COPE**. We used the BRIEF COPE inventory as a measure of coping strategies [20]. This inventory has 28 items and consists of 14 subscales—2 items per subscale. Each of the subscales measures a different coping mechanism, namely self-distraction, active coping, denial, substance use, use of emotional support, use of instrumental support, behavioural disengagement, venting, positive reframing, planning, humour, acceptance, religion, and self-blame. Examples of items include “I've been taking action to try to make the situation better” (active coping) and “I've been giving up the attempt to cope” (behavioural disengagement). The responses were anchored on 4-point likert scales, ranging from 1 (*not at* all) to 4 (*very* much). We asked participants their general strategies when they cope with their daily stress (rather than specific stressful events).

There exist many other widespread measures of coping strategies applicable to the general populations, including the Ways of Coping [73], the Coping Strategies Inventory [74], Multidimensional Coping Inventory [75], Coping Response’s Inventory [76], the COPE Inventory [77], and so on. We chose Brief COPE inventory because (a) it is a shortened version of the COPE Inventory when participant response burden is a considering factor, (b) the author recommended that researchers use the Brief COPE flexibly and creatively [20], and (c) researchers have been regularly using Brief COPE in an exploratory analysis to determine empirically how the data from their sample are to be analysed [78,79]. We did the subscale level factor analysis which was encouraged by the recommendations of the inventory’s author [20]. We conducted a factor analysis to determine the way in which the scores from the 14 coping subscales were structured in our data. This method is in line with many extant studies which extract 2–5 factors based on their samples [see review, 69].

**Trait empathy**. We used the 7-item empathic concern subscale and the 7-item perspective taking subscale from the Interpersonal Reactivity Index (IRI) [11]. IRI was designed to measure specific components of generic empathy, reflecting Davis’ conceptualization of empathy as a multidimensional construct involving both cognitive and affective processes. Guided by our hypothesis, we only focused on these two subscales in IRI. The empathic concern subscale is designed to measure the capacity to experience feelings of compassion, warmth, and concern in response to other people, whereas perspective taking subscale measures the individuals’ cognitive tendency of placing themselves in the position of others, and then adopting their psychological viewpoint [61].

Sample items from the empathic concern subscale are “I often have tender, concerned feelings for people less fortunate than me” and “Sometimes I don’t feel very sorry for other people when they are having problems. (reversed item)” Sample items from the perspective taking subscale are “I try to look at everybody’s side of a disagreement before I make a decision” and “I sometimes try to understand my friends better by imagining how things look from their perspective.” Each item was answered on a 5-point scale ranging from 0 (*not true of me at all*) to 4 (*frequently true of me*).

**Subjective SES**. We employed the MacArthur scale of subjective SES as a measure of individuals’ SES [37]. Participants were instructed to look at a picture of a ladder and were presented with the following: “Think of this ladder as representing where people stand in the United States. At the top of the ladder are the people who are the best off—those who have the most money, the most education, and the most respected jobs. At the bottom are the people who are the worst off—who have the least money, least education, and the least respected jobs or no job. The higher up you are on this ladder, the closer you are to the people at the very top and the lower you are, the closer you are to the people at the very bottom. Where would you place yourself on this ladder?” (10-point scale). We adapted the instruction to our UK sample for Study 5 and replaced ‘United States’ by ‘United Kingdom’.

### Results

#### Exploratory factor analysis

To examine the factor structure of the COPE questionnaire, factor analysis with varimax rotation was conducted on the 14 subscales. We chose to build the factors using subscales following the development of the COPE questionnaire [20] and many other studies (see [80] for a review). Cattell’s Scree Test suggested 2, 3 or 4 factors and we chose the 3 factors model, because the 4-factor model yielded more double-loadings greater than 0.3, and the 2-factor model generated factors in which the items of the two factors disagreed with items of the two factors in other studies. The selection of 3 factors was also supported by Optimal Coordinates, Parallel Analysis, Very Simple Structure (VSS) Complexity 2. Specifically, results of Horn’s Parallel Analysis suggested retaining 3 factors because the eigenvalue from real data was larger than the eigenvalue from the random data (see S1 Fig). VSS complexity 2 achieved a maximum of 0.74 with 3 factors.

Three factors also matched previous’ factor analysis results [21,22,81]. In our factor analysis, four subscales—venting, humor, religion and self-distraction—were dropped from the model because their loadings were lower than .40. Stevens (1992) suggested using a .40 cut-off loading, irrespective of sample size, for interpretative purposes [82], and the model selected with cut-off .40 loadings reached the best model fit for Studies 2–5. How removal of the four subscales changed loadings, factor structure and model fit is reported in the supplementary materials (Tables A-E in S1 File). 10 out of 14 subscales entered the final model, explaining 54% of the total variance. The chi-square statistic was 82.56 on 18 degrees of freedom, *p* < .05. It is well documented that the chi-square test is very sensitive to sample size[83], we therefore did not reject the model based on chi-square results.

In the final model (see Table 1), four subscales—active coping, positive reframing, planning, and acceptance—loaded highest on the first factor. The second factor was composed of 2 subscales: use of instrumental support and use of emotional support. The third factor was composed of four subscales: denial, substance use, behavioural disengagement, and self-blame. We named the three factors adaptive coping, use of social support (or social support for short), and maladaptive coping, respectively. However, it is worth noting that the strategies in the factor which we named “adaptive” or “maladaptive” copings may not always be adaptive or maladaptive respectively, as discussed in the introduction.

**Table 1:** Three factors for coping and subscales in each coping style.

Factor	Subscale	Eigenvalue and Adjusted *R*^2^
1. Adaptive coping	Active coping	Eigenvalue = 3.42; *R*^*2*^ = .22
	Positive reframing	
	Planning	
	Acceptance	
2. Social support	Use of instrumental support	Eigenvalue = 1.41; *R*^*2*^ = .17
	Use of emotional support	
3. Maladaptive coping	Denial	Eigenvalue = 1.20; *R*^*2*^ = .15
	Substance use	
	Behavioural disengagement	
	Self-blame	

#### Main analyses

As a note, in all the results presented below (Studies 1–4), “empathy” refers to “empathic concern” and not “perspective taking”. Indeed, we found no interaction effect between perspective taking and SES on any coping strategies. Results related to perspective taking can be found in the supplementary materials (S1–S4 Files).

Descriptive statistics for Study 1 are presented in Table 2. Hierarchical regression analyses were conducted to determine the effect of SES and empathy on adaptive coping, social support, and maladaptive coping, as shown in Table 3. We centered subjective SES and trait empathy. Trait empathy was entered in the first block. The second block contained both trait empathy and subjective SES. Then the interaction term of trait empathy and subjective SES was entered in the third block. As shown in the first and second regression models, both empathy and SES positively predicted adaptive coping and social support, and negatively predicted maladaptive coping. More importantly, as suggested by the third model, the interaction between empathy and SES significantly predicted maladaptive coping, but not adaptive coping nor social support. We display both standardized (β) and non-standardized (*b*) effect size in the tables.

**Table 2:** Study 1 participants’ means and standard deviations on the measure of SES, empathy, and coping strategies.

	SES	Empathy	Adaptive coping	Social support	Maladaptive coping
*M*	4.62	2.87	3.16	2.72	1.86
*SD*	1.67	.75	.47	.73	.55

**Table 3:** Hierarchical regression models predicting coping strategies in Study 1.

A. Model for adaptive coping															
	β	*b*	*SE*	*t*	95% CI	β	*b*	*SE*	*t*	95% CI	β	*b*	*SE*	*t*	95% CI
Empathy	.12	.16	.03	4.78***	.09, .22	.12	.16	.03	4.99***	.10, .23	.12	.16	.03	5.03***	.10,.23
SES						.08	.05	.01	3.30***	.02, .08	.08	.05	.01	3.43***	.02,.08
Empathy× SES											.03	.02	.02	1.26	-.01,.06
*R*^2^	.06					.09					.10				
Adjusted *R*^2^	.06					.09					.09				
*F*	22.88***					17.23***					12.03***				
B. Model for social support															
	β	*b*	*SE*	*t*	95% CI	β	*b*	*SE*	*t*	95% CI	β	*b*	*SE*	*t*	95% CI
Empathy	.14	.18	.05	3.54***	.08, .29	.15	.20	.05	3.94***	.10, .30	.15	.20	.05	3.98***	.10, .30
SES						.21	.13	.02	5.69***	.08, .17	.22	.13	.02	5.80***	.09, .17
Empathy× SES											.05	.04	.03	1.30	-.02, .10
*R*^2^	.04					.12					.12				
Adjusted *R*^2^	.03					.12					.12				
*F*	12.56***					23.02***					15.94***				
C. Model for maladaptive coping															
	β	*b*	*SE*	*t*	95% CI	β	*b*	*SE*	*t*	95% CI	β	*b*	*SE*	*t*	95% CI
Empathy	-.09	-.11	.04	-2.89**	-.19, -.04	-.09	-.12	.04	-3.12**	-.20,-.04	-.09	-.12	.04	-3.19**	-.20, -.05
SES						-.12	-.07	.02	-4.11***	-.11, -.04	-.13	-.08	.02	-4.33***	-.11, -.04
Empathy× SES											-.06	-.05	.02	-2.00*	-.09, .00
*R*^2^	.02					.07					.08				
Adjusted *R*^2^	.02					.07					.07				
*F*	8.33**					12.82***					9.96***				

* *p* < .05,

** *p* < .01,

*** *p* < .001

**Fig 1:**
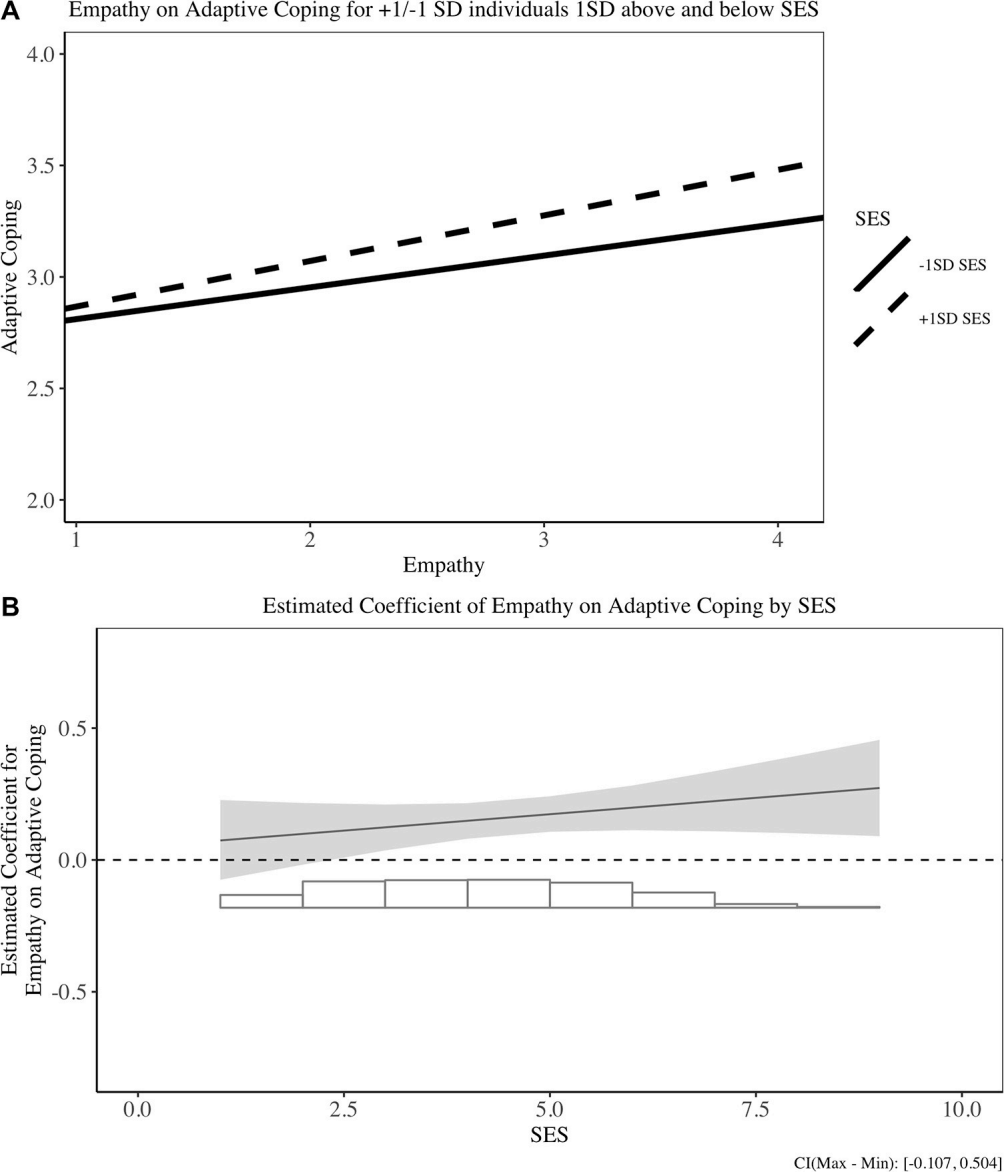
Relationship between empathy and adaptive coping in Study 1. (A) Simple slope result for +/- 1SD SES individuals on adaptive coping. (B) Estimated coefficient of empathy on adaptive coping for different SES individuals.

**Fig 2:**
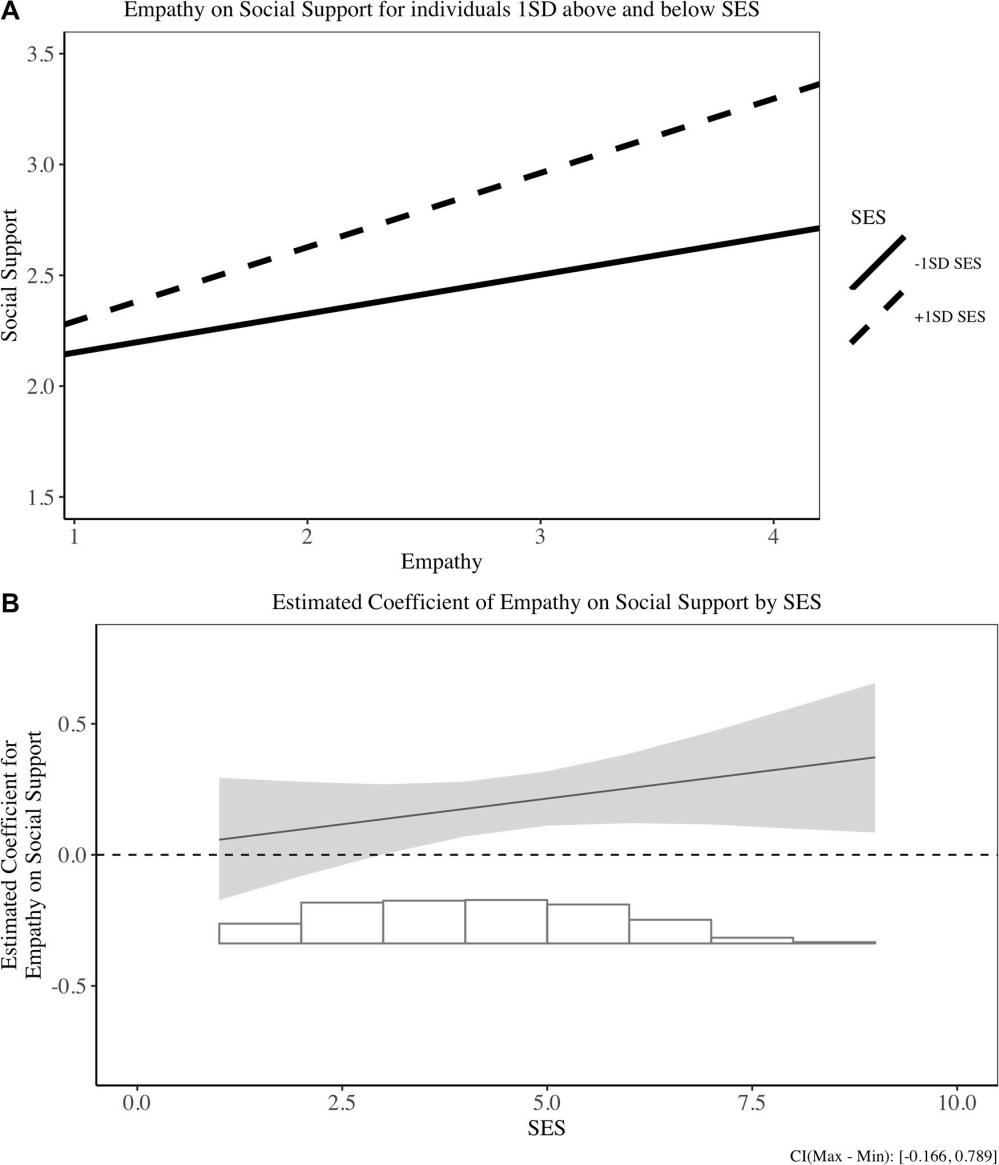
Relationship between empathy and social support in Study 1. (A) Simple slope result for +/- 1SD SES individuals on social support. (B) Estimated coefficient of empathy on social support for different SES individuals.

**Fig 3:**
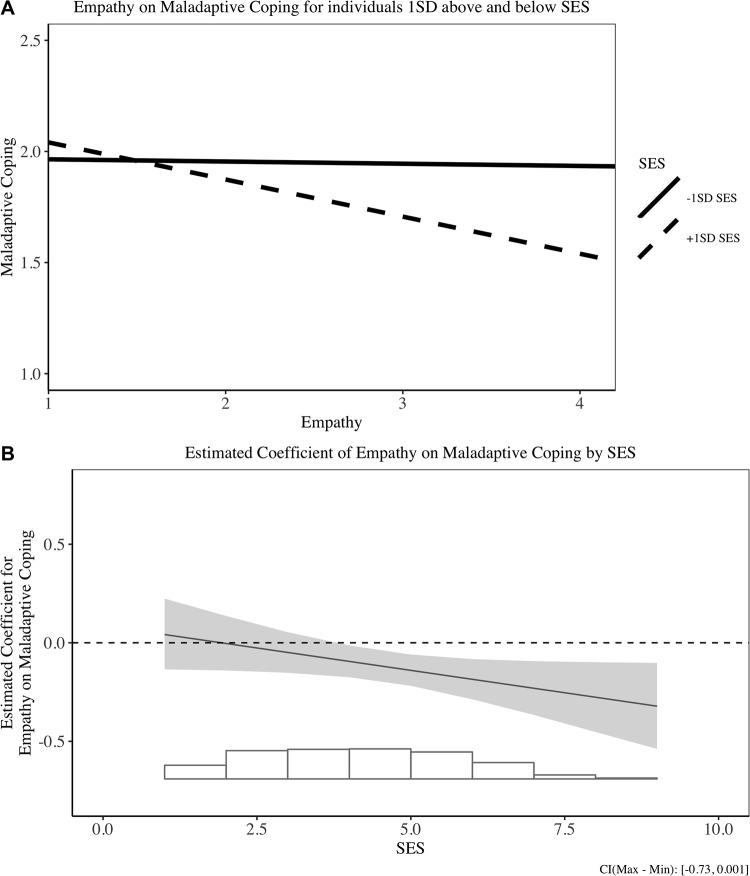
Relationship between empathy and maladaptive coping in Study 1. (A) Simple slope result for +/- 1SD SES individuals on maladaptive coping. (B) Estimated coefficient of empathy on maladaptive coping for different SES individuals.

Subsequently, we conducted simple slope analyses to assess the effect of empathy on coping strategies at different levels of SES (i.e., +/- 1 *SD* of the mean) [84]. The simple slope analyses suggest that empathy negatively predicted the use of maladaptive coping strategies for high SES people, *b* = -.20, 95% CI [-.31, -.09], *SE* = .06, *t*(335) = -3.63, *p* < .001, but not for low SES people, *b* = -.05, 95% CI [-.15, .06], *SE* = .05, *t*(384) = -.87, *p* > .1 We display two types of figures to visualize the interaction results. Figs 1(A)–3(A) display the interaction results between empathy and coping strategies for individuals with +/- 1*SD* of mean SES, and Figs 1(B)–3(B) illustrate a more continuous influence of empathy on coping for different SES levels. We plot the effects for all three types of coping for comprehensiveness. These findings support the rich-protection hypothesis: Empathy trait protects the rich from using less maladaptive coping strategies.

## Study 2

### Method

#### Participants and procedure

Another 400 participants from the United States were recruited via Amazon MTurk for this study. After removing participants who had duplicated entries and those who had incomplete cases, 394 participants (Male = 196, *M*_age_ (*SD*) = 34.70 (11.20)) were used in the final analysis. Participants followed the same procedures as in Study 1.

### Results

#### Confirmatory factor analysis

First, we validated the factor structure of coping strategies using confirmatory factor analysis (CFA). The three-factor model fit the data well, *CFI* = 0.96, *RMSEA* = 0.08, *SRMR* = 0.06. Though not perfect (*RMSEA* = .08, at the upper limit), the CFA results confirmed the factorial validity of coping strategies among MTurk users in the United States. Participants’ means and standard deviations on the measure of SES, empathy, and coping strategies were listed in the supplementary materials (Table A in S2 File).

#### Main analyses

**Fig 4:**
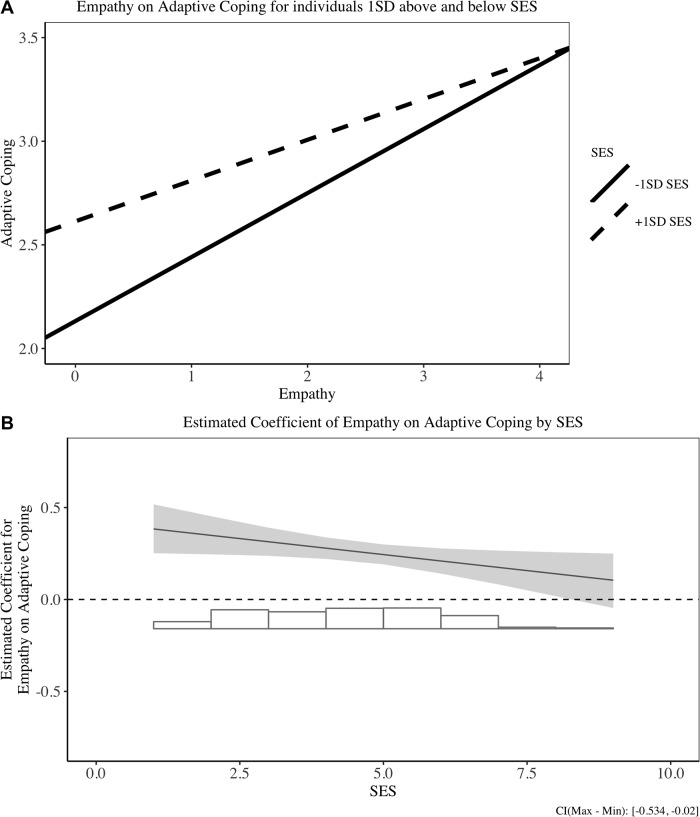
Relationship between empathy and adaptive coping in Study 2. (A) Simple slope result for +/- 1SD SES individuals on adaptive coping. (B) Estimated coefficient of empathy on adaptive coping for different SES individuals.

We then applied the same analysis steps as in Study 1 to test the relationship between empathy, SES and coping strategies. All results are presented in the supplementary materials (Table B in S2 File). In line with Study 1, empathy and SES both positively predicted adaptive coping and social support, and negatively predicted maladaptive coping. However, contrary to Study 1, we found an interaction effect of empathy and SES on adaptive coping, not on social support and adaptive coping. Interestingly, this finding supports the poor-protection hypothesis: empathy helps the poor to use more adaptive coping. Fig 4(A) displays the interaction results between empathy and adaptive coping for individuals with +/- 1*SD* of mean SES, and Fig 4(B) illustrates a more continuous influence of empathy on adaptive coping for different SES levels.

## Study 3 and Study 4

In Study 2, we verified the factor structure of coping, and replicated the empathy—coping relationship in Study 1: Empathy was positively related to the use of adaptive coping and social support, and was negatively related to the usage of maladaptive coping. However, in Study 2 we could not fully replicate the empathy × SES interaction found in Study 1. This might be due to the problems of MTurk participant sample as the literature suggests a lower reliability for studies conducted on Amazon MTurk [72]. To avoid this problem, we replicated the same study using two larger samples from survey panels Tellwut and Cint. The three-factor model fits the data well in both Studies 3 and 4. For Study 3, *CFI* = 0.96, *RMSEA* = 0.07, *SRMR* = 0.04. For Study 4, *CFI* = 0.97, *RMSEA* = 0.07, *SRMR* = 0.04.

Consistent with the results in Studies 1 and 2, empathy was positively related to adaptive coping and social support, and negatively related to maladaptive coping strategies in both Studies 3 and 4. The empathy × SES interaction on adaptive coping and maladaptive coping supported both the poor-protection hypothesis and rich-protection hypothesis: the empathy—adaptive coping positive relationship was stronger for lower SES individuals, and the empathy—maladaptive coping negative relationship was strongly for higher SES individuals.

Detailed results of Studies 3 and 4 are reported in the supplementary materials (S3 and S4 Files).

We summarise the main effect, interaction and simple slope analysis results from Study 1–4 in Table 4.

**Table 4:** Results summary from Studies 1–4.

Study	Basic info	outcome	Adaptive coping	Social support	Maladaptive coping
Study 1	Mturk	Empathy’s main effect	+^b^	+	-
	*N* = 339	Interaction	NS^a^	NS	- ^c^
	Male = 146 (43%)	Simple slope effect on -1*SD* SES	NA^e^	NA	NS
	Age = 34.14 (*SD* = 8.98)	Simple slope effect on +1*SD* SES	NA	NA	-
Study 2	Mturk	Empathy’s main effect	+	+	-
	*N* = 394	Interaction	-	NS	NS
	Male = 196 (50%)	Simple slope effect on -1*SD* SES	++ ^d^	NA	NS
	Age = 34.70 (*SD =* 11.20)	Simple slope effect on +1*SD* SES	+	NA	NS
Study 3	Tellwut	Empathy’s main effect	+	+	-
	*N* = 1477	Interaction	NS	-	-
	Male = 307 (21%)	Simple slope effect on -1*SD* SES	NA	++	-
	Age = 22.48 (*SD =* 13.15)	Simple slope effect on +1*SD* SES	NA	+	—
Study 4	Cint	Empathy’s main effect	+	+	-
	*N* = 1132	Interaction	-	-	-
	Male = 537 (47%)	Simple slope effect on -1*SD* SES	++	+	NS
	Age = 41.06 (*SD =* 12.55)	Simple slope effect on +1*SD* SES	+	NS	-

^a^. NS = Not Significant

^b^. For main effect results, ‘+’ means empathy had a positive main effect on coping; ‘-’ means empathy had a negative main effect on coping

^c^. For interaction results, ‘+’ means there was a positive interaction effect between empathy and SES on coping; ‘-’ means there was a negative interaction effect between empathy and SES on coping

^d^. For simple slope analysis results, ‘++’ or ‘—’ means that empathy’s influence on coping was larger for this SES group than the other, even when both have the same trend

^e^. NA = Not Applicable. No simple slope analysis was conducted if there was no interaction effect

## Study 5

### Method

#### Participants

Participants were recruited from the Cambridge BioResource Center which has a panel of over 15,000 volunteers who donated their DNA via blood or saliva sample and consented to be approached for research studies. Four hundred and thirty participants took part in the survey, but 70 cases did not provide enough information for analyses and thus were removed from the final analyses, making the final sample size 360 Caucasian white participants (Male = 138, 1 whose gender was not reported), *M*_age_ (*SD*) = 55.63 (11.16), age ranged 23–81. Participants’ OXTR receptor genotypes were nearly evenly distributed, GG = 118, AG = 122, AA = 120—this was purposeful in order to compare them.

#### Procedure

Participants were recruited via emails sent by the Cambridge BioResource Centre, those who showed interest in taking part consented electronically and completed the 20-min study online. This study received ethical approval from the University of Cambridge Research Ethics Committee. The questionnaires were identical to the 4 previous studies.

**Confirmatory Factor Analysis**. Compared to Studies 1–4, Study 5 was conducted in the UK. To confirm the validity of our three-factor model, we ran another CFA. The three-factor model fit the data well, *CFI* = .94, *RMSEA* = .07, *SRMR* = .05. The CFA results confirmed the factorial validity of coping strategies among a community sample in the UK.

**Analysis Plan**. In previous OXTR research conducted in western countries, given the limited number of AA individuals, many studies combine AG and AA into the same gene group. Here, we were able to recruit in equal numbers across the genotypes, making it possible to analyse GG, AG and AA as three different gene types. We dummy-coded the oxytocin receptor genotype. We first ran a linear regression to examine whether there was a genetic difference in the level of adaptive coping, social support, and maladaptive coping. We used the dummy-coded genotype as predictors (for example, we put genotype GG and genotype AG in the model, with genotype AA as the reference group) and coping strategies as outcomes.

Second, we conducted a series of hierarchical regression analyses to test the gene × SES interaction hypothesis that SES would moderate the relationship between OXTR (IV) and usage of coping strategies (DV). SES was normally distributed in this sample, so we did not further transform it, but we centered it before the hierarchical regression analysis. At Step 1, OXTR and SES were entered as predictors, coping strategies entered as outcomes. At Step 2, OXTR, SES, and the interaction term of OXTR and SES were entered as predictors. The moderator effect was indicated by a significant interaction of OXTR genotype and SES on individuals’ coping strategies. Similar to Studies 1–4, we conducted the three regression analyses for adaptive coping, social support, and maladaptive coping separately.

Lastly, for the models with a significant result on the interaction terms, we conducted simple slope analyses for individuals with low SES (1*SD* lower than mean SES) and for individuals with high SES (1*SD* higher than mean SES), to examine what coping strategies are used by both high and low SES individuals with AA, AG and GG genotypes.

## Results

Participants’ descriptive results on SES and coping strategies are shown in Table 5.

**Table 5:** Study 5 participants’ means and standard deviations on the measure of SES, empathy, and coping strategies.

	SES	Adaptive coping	Social support	Maladaptive coping
*M*	6.00	3.33	2.86	1.77
*SD*	1.46	.40	.76	.50

**Fig 5:**
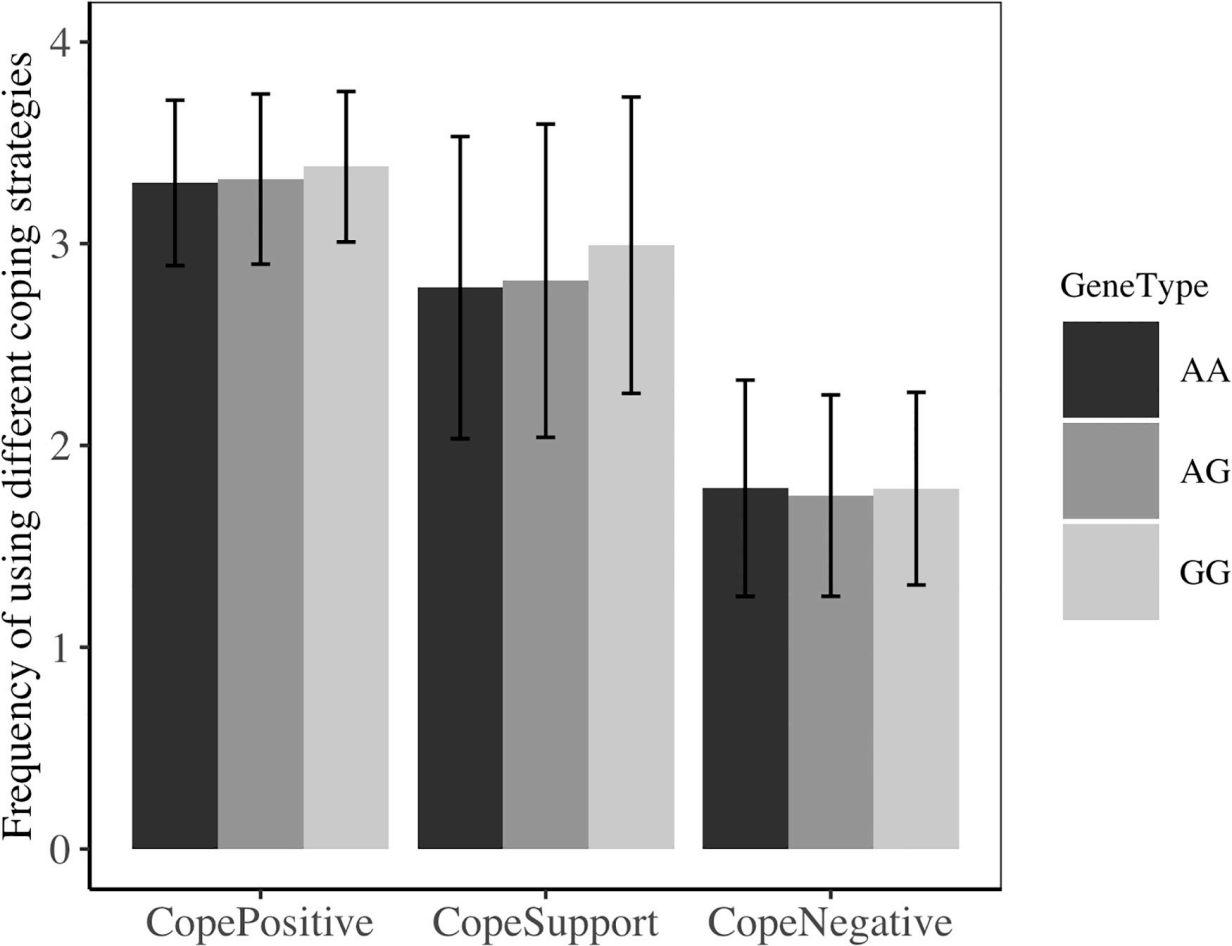
Participants’ usage of coping strategies across different genotypes in Study 5 (with SD).

We first tested whether individuals with GG, AG and AA genotypes used different coping strategies. Our models suggested that there was no difference between GG, AG, and AA individuals in predicting the usage of adaptive coping (GG vs AG, GG vs AA, AG vs AA, all *p*s > .5) nor maladaptive coping (GG vs AG, GG vs AA, AG vs AA, all *p*s > .50); however for social support, we found that, GG (*Mean* = 2.99, *SE* = .10) genotype individuals used significantly more social support than AA (*Mean* = 2.78, *SE* = .07) genotype individuals, *t*(357) = 2.15, *p* = .03, and marginally more than AG individuals (*Mean* = 2.82, *SE* = .10), *t*(357) = 1.81, *p* = .07. We further discussed the results in the discussion part. Participants’ usage of coping strategies in different genotypes is shown in Fig 5.

### Use of adaptive coping

**Fig 6:**
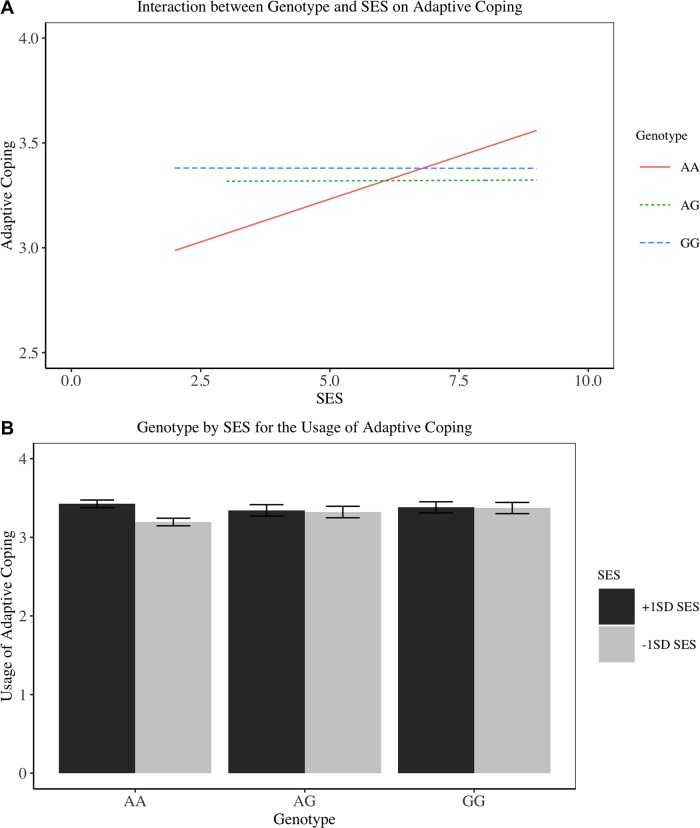
Interaction between genotype and SES on adaptive coping in Study 5. (A) Line plot. (B) Bar plot with standard deviation.

As Table 6 shows, there was no effect of genotype or SES on the usage of adaptive coping. However, there was an interaction between genotype and SES. Simple slope analyses revealed that for individuals with lower SES (1 *SD* below the mean SES), those with GG genotype use more adaptive coping strategies than those with AA genotype, *b* = .19, 95% CI [.04, .33], *SE* = .07, *t*(349) = 2.56, *p* = .01. This effect was not found for higher SES individuals (*Mean*_AA_ = 3.43, *Mean*_AG_ = 3.34, *Mean*_GG_ = 3.38, all *p*s > .10). The interaction is visually displayed in Fig 6. Since there are two interaction terms between SES and the two dummy variables, SES × GG and SES × AG, we also tested the robustness of the interaction results by correcting for multiple comparisons in the model. We used α = 0.025 for the significance level of the two interaction terms. The SES × GG and SES × AG interaction terms are statistically marginally significant (*p =* .026) and statistically significant (*p =* .024), respectively. We also provided the standardized coefficient of the model, however, since the genotype was dummy coded, the standardized coefficient should be interpreted with caution.

**Table 6:** Hierarchical regression results for adaptive coping (with AA genotype as reference).

	Step 1					Step 2					Difference between Step 1 and Step 2
	β	*b*	*SE*	*t*	95% CI	β	*b*	*SE*	*t*	95% CI	
SES	.04	.03	.02	1.75	.00, .06	.12	.08	.03	3.12**	.03, .13	
GG	.08	.08	.05	1.45	-.03, .18	.07	.07	.05	1.24	-.04, .17	
AG	.01	.01	.05	0.20	-.09, .11	.01	.01	.05	0.11	-.10, .11	
SES × GG						-.12	-.08	.04	-2.23*	-.16, -.01	
SES × AG						-.12	-.08	.04	-2.26*	-.15, -.01	
*R*^2^	.02					.03					
Adjusted *R*^2^	.01					.02					
*F*	1.86					2.45*					3.31*

* *p* < .05,

** *p* < .01,

*** *p* < .001

### Use of social support

When considering genotype, SES and their interaction on the use of social support, we found no effect of SES on using social support, and no genotype × SES interaction on social support. However, as reported above in the genotype–coping regression, we found a main effect of genotype such that individuals with GG genotype used more social support than AA individuals. Results are reported in the supplementary materials (S5 File).

### Use of maladaptive coping

**Fig 7:**
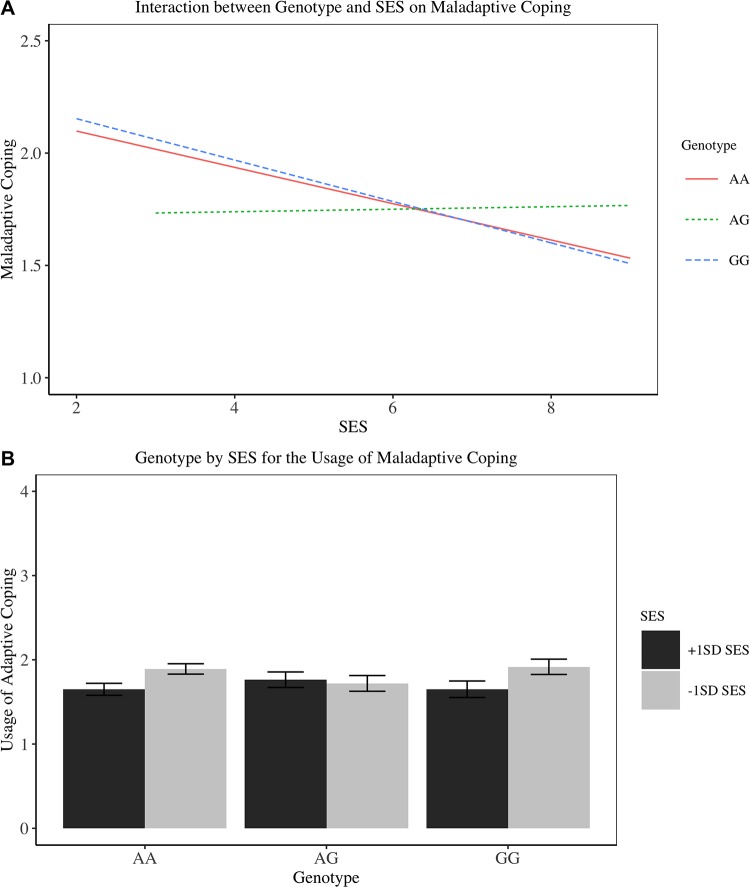
Interaction between genotype and SES on maladaptive coping in Study 5. (A) Line plot. (B) Bar plot with standard deviation.

As seen in Table 7, there was no effect of genotype on the usage of maladaptive coping. However, there was a negative relationship between SES and use of maladaptive coping, suggesting that higher SES individuals were less likely to use maladaptive coping strategies (results in Step 1). There was also a genotype × SES interaction–as hypothesised. Simple slope analyses revealed that for individuals 1*SD* below the mean SES, GG individuals were marginally more likely to use maladaptive coping compared to AG individuals (*Mean*_AG_ = 1.74), *b* = .18, 95% CI [-.01, .36], *SE* = .10, *t*(349) = 1.86, *p* = .06. The interaction for maladaptive coping is visually displayed in Fig 7.

**Table 7:** Hierarchical regression results for maladaptive coping.

	Step 1					Step 2					Difference between Step 1 and Step 2
	β	*b*	*SE*	*t*	95% CI	β	*b*	*SE*	*t*	95% CI	
SES	-.08	-.05	.02	-2.86**	-.09, -.02	-.12	-.08	.03	-2.46*	-.15, -.02	
GG	.01	.01	.07	0.08	-.12, .13	.01	.01	.07	.15	-.12, .14	
AG	-.02	-.02	.07	-0.24	-.14, .11	-.02	-.02	.07	-.38	-.15, .10	
SES × GG						-.02	-.01	.05	-.25	-.10, .08	
SES × AG						.13	.09	.04	1.93	.00, .17	
*R*^2^	0.02					.04					
Adjusted *R*^2^	0.02					.03					
*F*	2.88*					2.93*					2.96 (*p* = .053)

* *p* < .05,

** *p* < .01

Table 8 summarised the results from Study 5.

**Table 8:** Results summary from Study 5.

Study	Basic info	outcome	Adaptive coping	Social support	Maladaptive coping
Study 5	Cambridge BioResource	Gene on coping strategies	NS^a^	GG > AA; GG > AG (marginally)	NS
	*N* = 360 (GG = 118, AG = 122, AA = 120)	Gene × SES interaction on Coping	GG vs AA, AG vs AA	NS	AG vs AA, AG vs GG
	male = 138	Simple slope effect on -1*SD* SES	GG individuals use more adaptive coping than AA individuals	NS	AG individuals use less maladaptive coping than GG and AA individuals
	Age = 55.63 (*SD* = 11.16)	Simple slope effect on +1*SD* SES	NS	NS	NS

^a^. NS = not significant

## Discussion

### Summary of our findings

In the present research, we aimed to explore the relationship between trait empathy and general coping strategies; in addition, we examined how individuals’ subjective SES moderated the relationship between empathy and coping. We tested our hypotheses in five studies across nearly 4000 participants in the United States and the United Kingdom, using both questionnaires and genotype analysis. In Studies 1–4 we replicated the same procedure across different recruitment platforms to examine the stability of the results. Participants reported their subjective SES, empathy and their coping strategies when dealing with daily stress. In Study 5, we asked individuals with oxytocin receptor gene OXTR rs53576 genotypes GG, AG, and AA to report their subjective SES and coping strategies, then tested whether the potential biological antecedent of empathy would also have an influence on coping strategies. Results were mixed. We consistently found that having higher empathic concern was related to the usage of more adaptive coping strategies (as measured with the active coping, positive reframing, planning and acceptance suggested by our factor analysis sub-scales, which we identified in our factor analysis) and social support (instrumental and emotional support), as well as using fewer maladaptive coping strategies (here denial, substance use, behavioural disengagement, and self-blame). We also found that the influence of trait empathy on the choice of coping strategies differed between the relatively poor and relatively rich. In Studies 1–4, both the *poor-protection* and *rich-protection* hypotheses held. In particular, for adaptive coping, the poor tended to benefit more from being high in empathic trait—supporting the *poor-protection* hypothesis. For maladaptive coping however, the rich tended to benefit the most from being high in empathic trait—supporting the *rich-protection* hypothesis. It is important to note, however, that these interactions were inconsistent across studies—in some studies, they would result in highly significant effects, whereas in others they would only be trending effects. In Study 5, we found that individuals with OXTR rs53576 polymorphism GG genotype were more likely to use social support than AG (marginally) and AA individuals. This was in line with previous research [85]. We also found a genotype by SES interaction on adaptive coping and maladaptive coping, which was consistent with Studies 1–4. The bullet points below summarise our main findings:

- Individuals with high empathic concern use more adaptive coping strategies and social support, and fewer maladaptive coping strategies compared with individuals with low empathy;
- We found support for the *poor-protection hypothesis* for adaptive coping strategies: the poor benefited the most from empathic concern as poor individuals high (vs low) in empathic concern used more adaptive coping strategies;
- We also found support for the *rich-protection hypothesis* for maladaptive coping strategies: the rich tend to benefit more from empathy as rich individuals high (vs low) in empathic concern used less maladaptive coping strategies;
- Individuals with the OXTR rs53576 genotype GG (suggested by meta-analysis and population studies to be the antecedent of trait empathy) used more social support compared to individuals with the AG and AA genotypes;
- In individuals with lower SES, those with the GG genotype used more adaptive strategies than those with the AA genotype, supporting the *poor-protection hypothesis*.

The inconsistency in the interactions deserves special attention. Overall, the high RMSEA across all models, in combination with the significant chi-square indicates that model fit is good but less than optimal; this may have partly contributed to the inconsistencies in the interactions. Across five studies, only two studies showed an empathy by subjective SES interaction for the usage of *social support*. This might be due to the factor itself. There were only two subscales in the social support factor. Even though Harvey et al. [86] suggested that at least four items per scale are needed to test the homogeneity of items within each latent construct, other researchers recently also suggested that it is possible to retain a factor with only two items if the items are highly correlated (i.e., *r* > .70) and relatively uncorrelated with other variables [87]. In our sample in Study 1, the “use of emotional support” subscale and “use of instrumental support” subscale were highly correlated, *r* > .80. However, this might still have partly contributed to the few interaction results on social support. For the interaction results on adaptive coping and maladaptive coping, we found significant interactions in Studies 2 and 4 for adaptive coping, and interactions in Studies 1, 3, and 4 for maladaptive coping. In Studies 1–2, we recruited participants from Amazon MTurk. Even though Amazon MTurk has been popular among social scientists, researchers have pointed out the potential problems with these samples, such as the fact that 10% of workers are responsible for completing 41% of tasks, and that more experienced workers are more familiar with classic paradigms within behavioural sciences [88,89], suggesting that their prior experiences in taking part in similar studies before may influence their responses in new research studies. Because we used a relatively popular measure of trait empathy, it may have partly contributed to the inconsistent results between Studies 1 and 2. For Study 3, we did not find an interaction between empathy and SES on adaptive coping, but the interaction for social support and maladaptive coping were the same as in Study 4. Participants in Study 3 were recruited from the survey panel Tellwut; however, the average age (around 23) was significantly lower than those recruited from MTurk (around 35) and Cint (around 41). The gender imbalance may also be a problem in Study 3 (21% men, compared to roughly 50% in the other 3 studies), even though controlling for gender and age did not change the outcomes.

The questionnaire results in Studies 1–4 suggest that there was indeed a relationship between empathy and people’s usage of coping strategies; therefore, in Study 5, we intended to examine whether there was a biological foundation under this relationship. We chose to test whether the oxytocin receptor gene rs53576 polymorphism, suggested to relate to one’s trait empathy level, would influence individuals’ coping strategies. Individuals with different genotypes did not differ on the usage of adaptive coping or maladaptive coping; however, individuals with GG genotype were more likely to use social support than individuals with AG and AA genotypes. This finding is in line with previous research that individuals with the GG or AG genotypes self-reported on a free recall task a higher tendency to seek social support relative to those with the AA genotype [85]. The link between OXTR GG alleles and use of social support as a coping strategy may be the result of being more socially engaging and socially interested [90]. We also found a genotype × SES interaction on adaptive coping and maladaptive coping. In line with Study 1 to 4, low SES individuals with rs53576 GG genotype were indeed more likely to use adaptive coping strategies compared to AA individuals, but there were no differences between genotypes amongst high SES participants. For maladaptive coping, even though there was a genotype × SES interaction (in line with Studies 1–4), participants with GG genotype responded similarly to AA genotype individuals (usually, participants with GG genotype perform more similarly to AG rather than AA individuals). In addition, lower SES participants with AG genotype used fewer maladaptive coping strategies than lower SES participants with GG and AA genotypes. This effect is inconsistent with the rest of the results in several ways. First, we found in Studies 1–4 that empathy was negatively related to maladaptive coping strategies, yet the rs53576 results follow a more inverted U-shaped curve (with AG being associated with lower levels of maladaptive coping compared to AA and GG). Second, we consistently found in Studies 1–4 a negative interaction between SES and empathy for maladaptive coping, but the interaction was positive between AG and SES. Given this inconsistency, there is clearly a need to replicate these results before making any strong conclusions about the effects of OXTR rs53576 on coping (and how SES moderates this link).

### Implications

The current research has several implications for the literature on coping and practices in life. This is the first project to directly explore the relationship between trait empathy and daily coping styles: our results consistently suggest that individuals high in empathy use more adaptive coping and social support, and less maladaptive coping. Besides theory contribution, our results may be useful for some clinical practices. Indeed, our results are in line with existing compassion-focused therapy practice and compassionate-mind training which train individuals on increasing empathy, compassion and mentalisation towards oneself [91,92].

Our results also suggested that empathy’s beneficial influence on coping was not uniform across all individuals; rather, empathy interacted with SES to influence coping. Both the *poor-protection* and *rich-protection* hypotheses yield accurate predictions, depending on the specific coping strategies considered. The specificity suggests different pathways for improving people’s coping toolkits. For instance, for lower SES people, we suggest it might be most prudent to focus on reducing their maladaptive coping strategies through interventions—since empathy on its own seems ineffective amongst this group for reducing maladaptive coping. On the other hand, for people with high SES, it would seem sensible to focus on the adaptive coping strategies through interventions, even among the highly empathic.

This research was also the first to explore whether individuals with different oxytocin receptor gene rs53576 polymorphism, with genotype GG, AG and AA, differ in using coping strategies. We found that individuals with GG genotype were more likely to use social support than individuals with AG and AA genotypes. Although there is some literature on the genetic basis of coping [93,94], there is no study, to the best of our knowledge, that explores how the genotype rs53576 influences individuals’ coping strategies. We suggest future research to investigate what it is about OXTR rs53576 that actually leads to the use of more social support.

### Considerations

It is worth emphasising that in the current examination, we only focused on one’s trait empathy rather than one’s motivation to empathise. While it is clear that individuals may have a stronger motivation to empathise in some situations than in others [95], we focused on the *ability* to empathise more globally. The measurement of empathy in the current study, “empathic concern”, in its nature may however be linked to some motivation, as it can be difficult to separate pure ability from motivation to concern for others, as noted by Gilbert’s work [96]. In the present research, we also did not look at people’s personality traits such as narcissism or trauma history, even though research has shown that it could influence empathy [40,97].

We also understand that subjective SES might not only function as a moderator in the relationship between trait empathy and coping strategies, but also directly influence coping styles (for instance, participants who use positive appraisal coping strategy might also estimate themselves as “better off”). In order to elucidate this interdependence, we ran a correlation between subjective SES and positive appraisal for Studies 1–5. Studies 1–4 showed a positive correlation between subjective SES and positive appraisal with all *r* less than .20, and Study 5 did not show a positive correlation (see supplementary materials S6 File for detailed results). The none-to-small correlations suggest that the usage of positive appraisal may have little relationship with people positively seeing themselves as “better off”. Moreover, we also ruled out the possibility of multicollinearity between subjective SES and empathic concern (see supplementary materials S6 File for detailed results).

As noted in literature, OXTR is not the only gene that is related to empathy, stress, and coping. For instance, research on the serotonin transporter promoter variant (5-HTTLPR) has found a significant interaction between 5-HTTLPR and both stressful life events and childhood maltreatment in the development of depression [98]. Individuals with two short alleles of 5-HTTLPR also report more personal distress and show higher levels of physiological responses in response to distressful films than individuals with long alleles [99]. However, since the current study is aimed at examining the relationship between trait empathy and coping strategies, we chose the potential biological antecedent of *empathy*, OXTR rs53576, rather than genes which are more related to stress and/or coping.

Last but not least, it is worth pointing out that OXTR rs53576 can be expressed phenotypically in different forms depending on the social environment. For instance, previous research has suggested that the expression of OXTR rs53576 is sensitive to cultural norms regarding emotion regulation. Emotional suppression is normative in East Asian cultures but not in American culture. For instance, among Americans, those with the GG genotype report using emotional suppression less than those with the AA genotype, whereas Koreans show the opposite pattern [100]. For an individual, it is therefore possible that different versions of OXTR rs53576 may get expressed and function differently depending on the environment this person is in. Given the complexity of epigenetics, our exploration of OXTR rs53576 and its relationship to coping strategies is only a small step in the exploration of what leads to better coping strategies.

### Limitations and future research

Our work has several limitations. First, in the selection of participants, we did not rule out participants who had mental and psychological illness, and we did not look at the specific environment our participants grew up in. As discussed in the introduction, lower SES individuals face more obstacles in life and are more likely to be stressed, anxious and depressed; the nature of the problems they encounter may also be different from their higher SES counterparts. Therefore, our results may be partly explained by participants’ anxiety and depression level, which we did not measure. Moreover, while we looked at the use of social support in our study, we did not measure participants’ relationship status or network situation. For example, people who have low SES may have more problematic relationships, may live alone, or may feel lonelier. All these factors could partly explain our findings. Finally, we did not look at the epigenetic influence on OXTR, which may have explained some of the inconsistencies in our results. Future research on this topic may consider controlling for participants’ mental and psychological illness and childhood trauma experiences.

A second limitation is related to the usage of Brief COPE questionnaire and what it actually measured in our samples. Although we followed the suggestions by the author of the scale, and our three factors were similar to other research using the same scale [21,22,81], what these three factors actually mean needs more examination. What fell in our “adaptive coping” category was active coping, positive reframing, planning and acceptance, with “active coping” and “planning” typical problem-focused coping strategies and generally more adaptive. Our “maladaptive coping” category included denial, substance use, behavioural disengagement, and self-blame, with “denial” and “self-blame” typical emotion-focused coping strategies. It should not be surprising that the effectiveness of emotion-focused coping depends on the particular form of emotion-focused strategy employed [77]. Nonetheless, the predominant view in the stress and coping literature is that emotion-focused coping processes are maladaptive [101]. Our results suggested an interaction between trait empathy (more specifically, empathic concern) and SES on “adaptive coping”, “maladaptive coping” and social support across studies, but we did not test whether the interaction was driven by certain “problem-focused” or “emotion-focused” coping strategies. One reason we did not do so was to avoid multiple comparison problems; however, future research should explore this. One may also suggest that in the strategies in our “adaptive” category require more interpersonal social processing while strategies in our “maladaptive” category can be regarded more as internal emotional processing. Future research should ask participants not only to self-report their coping style using a questionnaire, but also to give specific examples about how they apply these coping strategies in certain situations. Such data could help us better understand the mechanisms behind our findings.

Third, we only used empathic concern and perspective taking in the IRI questionnaire as a metrics for trait empathy, and we only used the BRIEF Cope questionnaire as a metrics for measuring individuals’ coping style. Because this study was an exploration of the relationship between empathy and coping strategies, single-measurement self-report metrics were justifiable. We did not measure the personal distress subscale of the IRI, meaning we could not test whether empathy as measured by personal distress would also cause stress through compassion fatigue. We suggest that future research should develop our findings using more than one scale to examine a larger range of coping strategies and aspects of empathy. We also suggest that future research should use multiple measures besides self-report, such as peer-evaluation and experience sampling.

A fourth limitation was that due to our sample size limitation for study 5, we could not directly build a link between OXTR rs53576 and trait empathy; that is, we could not draw a definite conclusion about the fact that individuals with GG alleles had higher trait empathy compared to individuals with AG alleles, or that individuals with AG alleles were more empathic than individuals with AA alleles. Our U-shape finding about AG individuals using less maladaptive coping than GG and AA individuals may actually suggest that the number of G allele does not linearly relate to trait empathy level. While meta-analysis and population studies suggest that OXTR rs53576 polymorphism might be the biological antecedent of trait empathy, our study suggest that we may need to be cautious. Indeed, it may not be the only biological antecedent of empathy. Therefore, our exploration of the genetic influences on coping was also preliminary. Even though we only partly replicated results from Studies 1–4 on both the main effect and the interaction effects on coping, it is important to note that many genetic studies fail to replicate [102]. In addition, genetic studies should ideally have sample sizes in the thousands rather than the hundreds. To solve this problem, future researchers may consider conducting a meta-analysis, taking stock of the available evidence, especially in domains with diverging sample sizes and contrasting outcomes [103].

Finally, future research is also needed to understand the mechanism behind the moderation effect and why empathy had different protection mechanisms for the lower vs. higher SES individuals.

To conclude, our work was the first one to explore how trait empathy influenced coping with daily stress, and how this relationship differed among the poor vs the rich. Results of this research are beneficial for healthcare, stress, well-being and burnout research areas, as well as for policy makers. Future researchers may explore the causal relationship between empathy and coping, as well as in a closer look at coping in different scenarios, cultures, etc.

## Supporting information

S1 File EFA and PT results in Study 1. (DOCX)

S2 File Supplementary results for Study 2. (DOCX)

S3 File Supplementary results for Study 3. (DOCX)

S4 File Supplementary results for Study 4. (DOCX)

S5 File Supplementary results for Study 5. (DOCX)

S6 File Considerations. (DOCX)

S1 Fig Illustration of parallel analysis results in Study 1. (PNG)

S2 Fig Relationship between empathy and social support in Study 2. (TIFF)

S3 Fig Relationship between empathy and maladaptive coping in Study 2. (TIFF)

S4 Fig Relationship between empathy and adaptive coping in Study 3. (TIFF)

S5 Fig Relationship between empathy and social support in Study 3. (TIFF)

S6 Fig Relationship between empathy and maladaptive coping in Study 3. (TIFF)

S7 Fig Relationship between empathy and adaptive coping in Study 4. (TIFF)

S8 Fig Relationship between empathy and social support in Study 4. (TIFF)

S9 Fig Relationship between empathy and maladaptive coping in Study 4. (TIFF)

S10 Fig Interaction between genotype and SES on social support in Study 5. (TIFF)
