# Supplementary material for: Caring helps: Trait empathy is related to better coping strategies and differs in the poor versus the rich
Source: PLoS One. 2019 Mar 27;14(3):e0213142. doi: 10.1371/journal.pone.0213142 (PMC6436718; doi:10.1371/journal.pone.0213142)
Supplement: S1 File — (DOCX) [file pone.0213142.s001.docx]

**Exploratory Factor Analysis for Study 1**

The selection of 3 factors was supported by Optimal Coordinates, Parallel Analysis, VSS Complexity 2. Parallel Analysis suggested 3 components to retain, please see S1 Fig.

**S1 Fig. Illustration of parallel analysis results**

We used a factor loading cut-off score of .40 in the current study. How removal of the four subscales changed loadings, factor structure and model fit is reported below:

Step 1: All 14 subscales are included in the factor analysis. The chi-square statistic is 259.55 on 52 degrees of freedom, p < .001.

**Table A. Factor Analysis Using All 14 Subscales**

|  | Factor1 | Factor2 | Factor3 |
| --- | --- | --- | --- |
| Active Coping | 0.80 |  | -0.31 |
| Positive Reframing | 0.56 |  |  |
| Planning | 0.88 |  | -0.21 |
| UseOfEmotionalSupport | 0.22 | 0.96 |  |
| UseOfInstrumentalSupport | 0.28 | 0.76 |  |
| Substance Use |  |  | 0.57 |
| Behavioral Disengagement | -0.22 |  | 0.75 |
| Self Distraction | 0.39 | 0.20 |  |
| Denial |  |  | 0.49 |
| Venting |  | 0.26 | 0.32 |
| Humor | 0.25 |  |  |
| Acceptance | 0.48 |  |  |
| Religion | 0.23 | 0.28 |  |
| Self Blame |  | -0.26 | 0.49 |
| SS loadings | 2.45 | 1.89 | 1.70 |
| Proportion Var | 0.18 | 0.14 | 0.12 |
| Cumulative Var | 0.18 | 0.31 | 0.43 |

Step 2: We removed “Humor” which had the lowest loading in Step 1. The rest 13 subscales entered the factor analysis. The chi-square statistic is 185.98 on 42 degrees of freedom, p < .001.

**Table B. Factor Analysis Using 13 Subscales (“Humor” removed)**

|  | Factor1 | Factor2 | Factor3 |
| --- | --- | --- | --- |
| Active Coping | 0.82 |  | -0.26 |
| Positive Reframing | 0.55 |  |  |
| Planning | 0.89 |  |  |
| UseOfEmotionalSupport | 0.20 | 0.97 |  |
| UseOfInstrumentalSupport | 0.27 | 0.77 |  |
| Substance Use |  |  | 0.56 |
| Behavioral Disengagement | -0.26 |  | 0.74 |
| Self Distraction | 0.36 | 0.22 |  |
| Denial |  |  | 0.48 |
| Venting |  | 0.27 | 0.33 |
| Acceptance | 0.46 |  |  |
| Religion | 0.22 | 0.28 |  |
| Self Blame | - | 0.25 | 0.49 |
| SS loadings | 2.40 | 1.93 | 1.62 |
| Proportion Var | 0.18 | 0.15 | 0.12 |
| Cumulative Var | 0.18 | 0.33 | 0.46 |

Step 3: We removed “Religion” which had the lowest loading in Step 2. The rest 12 subscales entered the factor analysis. The chi-square statistic is 137.64 on 33 degrees of freedom, p < .001.

**Table C. Factor Analysis Using 12 Subscales (“Humor” and “Religion” removed)**

|  | Factor1 | Factor2 | Factor3 |
| --- | --- | --- | --- |
| Active Coping | 0.83 |  | -0.25 |
| Positive Reframing | 0.55 |  |  |
| Planning | 0.90 |  |  |
| UseOfEmotionalSupport | 0.24 | 0.94 |  |
| UseOfInstrumentalSupport | 0.29 | 0.78 |  |
| Substance Use |  |  | 0.57 |
| Behavioral Disengagement | -0.28 |  | 0.72 |
| Self Distraction | 0.37 | 0.21 |  |
| Denial |  |  | 0.48 |
| Venting |  | 0.25 | 0.35 |
| Acceptance | 0.47 |  |  |
| Self Blame | - | 0.28 | 0.49 |
| SS loadings | 2.43 | 1.79 | 1.58 |
| Proportion Var | 0.2 | 0.15 | 0.13 |
| Cumulative Var | 0.2 | 0.35 | 0.48 |

Step 4: We removed “Venting”. The rest 11 subscales entered the factor analysis. The chi-square statistic is 105.62 on 25 degrees of freedom, p < .001.

**Table D. Factor Analysis Using 11 Subscales (“Humor”, “Religion” and “Venting” removed)**

|  | Factor1 | Factor2 | Factor3 |
| --- | --- | --- | --- |
| Active Coping | 0.81 |  | -0.28 |
| Positive Reframing | 0.55 |  |  |
| Planning | 0.90 |  |  |
| UseOfEmotionalSupport | 0.24 | 0.89 |  |
| UseOfInstrumentalSupport | 0.29 | 0.82 |  |
| Substance Use |  |  | 0.53 |
| Behavioral Disengagement | -0.23 |  | 0.81 |
| Self Distraction | 0.36 | 0.22 |  |
| Denial |  |  | 0.48 |
| Acceptance | 0.47 |  |  |
| Self Blame | - | 0.29 | 0.45 |
| SS loadings | 2.34 | 1.71 | 1.56 |
| Proportion Var | 0.21 | 0.16 | 0.14 |
| Cumulative Var | 0.21 | 0.37 | 0.51 |

Step 5: We removed “Self Distraction”. The final 10 subscales entered the factor analysis. The chi-square statistic is 82.56 on 18 degrees of freedom, p < .001.

**Table E. Factor Analysis Using 11 Subscales (“Humor”, “Religion”, “Venting” and “Self Distraction” removed)**

|  | Factor1 | Factor2 | Factor3 |
| --- | --- | --- | --- |
| Active Coping | 0.83 |  | -0.23 |
| Positive Reframing | 0.55 |  |  |
| Planning | 0.89 |  |  |
| UseOfEmotionalSupport | 0.23 | 0.91 |  |
| UseOfInstrumentalSupport | 0.28 | 0.81 |  |
| Substance Use |  |  | 0.51 |
| Behavioral Disengagement | -0.25 |  | 0.83 |
| Denial |  |  | 0.47 |
| Acceptance | 0.47 |  |  |
| Self Blame | - | 0.29 | 0.43 |
| SS loadings | 2.23 | 1.71 | 1.48 |
| Proportion Var | 0.22 | 0.17 | 0.15 |
| Cumulative Var | 0.22 | 0.39 | 0.54 |

**Study 1 PT results**

In Study 1, participants’ perspective taking (PT) scores ranged from 0 to 4, with mean score 2.77 and SD .67. Similar to empathic concern (EC), PT positively was related to adaptive coping (*b* = .26, *SE* = .04, *t*(337) = 7.27, *p* < .001, 95%*CI* [.19, .32]) and use of social support (*b* = .22, *SE* = .06, *t*(337) = 3.87, *p* < .001, 95%*CI* [.11, .34]), and was negatively related to maladaptive coping (*b* = -.20, *SE* = .04, *t*(337) = -4.73, *p* < .001, 95%*CI* [-.29, -.12]). When considering PT and SES together, the same direction preserved (*ps* < .001). However, there was no interaction between PT and SES on any coping strategies (*ps* > .1).
