# Supplementary material for: Caring helps: Trait empathy is related to better coping strategies and differs in the poor versus the rich
Source: PLoS One. 2019 Mar 27;14(3):e0213142. doi: 10.1371/journal.pone.0213142 (PMC6436718; doi:10.1371/journal.pone.0213142)
Supplement: S2 File — (DOCX) [file pone.0213142.s002.docx]

**Study 2**

Similar to Study 1, in Study 2, participants’ perspective taking (PT) scores ranged from 0 to 4, with mean score 2.61 and SD .80. Same as in Study 1, PT was positively related to adaptive coping (*b* = .30, *SE* = .03, *t*(393) = 10.28, *p* < .001, 95%*CI* [.24, .35]) and use of social support (*b* = .31, *SE* = .05, *t*(396) = 6.72, *p* < .001, 95%*CI* [.22, .40]), and was negatively related to maladaptive coping (*b* = -.16, *SE* = .04, *t*(392) = -4.62, *p* < .001, 95%*CI* [-.24, -.09]). When considering PT and SES together, the same direction preserved (*ps* < .001). However, there was no interaction between PT and SES on any coping strategies (*ps* > .1).

**Table A. Study 2 Participants’ Means and Standard Deviations on the Measure of SES, Empathy, and Coping Strategies**

|  | SES | Empathy | Adaptive coping | Social support | Maladaptive coping |
| --- | --- | --- | --- | --- | --- |
| *M* | 4.78 | 2.86 | 3.06 | 2.67 | 1.83 |
| *SD* | 1.60 | .88 | .52 | .78 | .58 |

**Table B. Hierarchical Regression Models Predicting Coping Strategies in Study 2**

| 1. Model for adaptive coping | | | | | | | | | | | | | | | |
| --- | --- | --- | --- | --- | --- | --- | --- | --- | --- | --- | --- | --- | --- | --- | --- |
|  | β | *b* | *SE* | *t* | 95% CI | β | *b* | *SE* | *t* | 95% CI | β | *b* | *SE* | *t* | 95% CI |
| Empathy | .22 | .25 | .03 | 9.36*** | .20, .31 | .22 | .26 | .03 | 9.64*** | .20, .31 | .22 | .25 | .03 | 9.54*** | .20, .31 |
| SES |  |  |  |  |  | .09 | .05 | .01 | 3.72*** | .03, .08 | .08 | .05 | .01 | 3.63*** | .02, .08 |
| Empathy× SES |  |  |  |  |  |  |  |  |  |  | -.05 | -.04 | .02 | -2.13* | -.07, .00 |
| *R*^2^ | .18 |  |  |  |  | .21 |  |  |  |  | .22 |  |  |  |  |
| Adjusted *R*^2^ | .18 |  |  |  |  | .21 |  |  |  |  | .21 |  |  |  |  |
| *F* | 87.62*** |  |  |  |  | 52.17*** |  |  |  |  | 36.61*** |  |  |  |  |
| 1. Model for social support | | | | | | | | | | | | | | | |
|  | β | *b* | *SE* | *t* | 95% CI | β | *b* | *SE* | *t* | 95% CI | β | *b* | *SE* | *t* | 95% CI |
| Empathy | .28 | .32 | .04 | 7.64*** | .24, .40 | .29 | .33 | .04 | 7.90*** | .25, .41 | .28 | .32 | .04 | 7.80*** | .24, .40 |
| SES |  |  |  |  |  | .14 | .09 | .02 | 3.80*** | .04, .13 | .14 | .09 | .03 | 3.74*** | .04, .13 |
| Empathy× SES |  |  |  |  |  |  |  |  |  |  | -.04 | -.03 | .03 | -1.22 | -.08, .02 |
| *R*^2^ | .13 |  |  |  |  | .16 |  |  |  |  | .16 |  |  |  |  |
| Adjusted *R*^2^ | .13 |  |  |  |  | .16 |  |  |  |  | .16 |  |  |  |  |
| *F* | 58.39 |  |  |  |  | 37.41 |  |  |  |  | 25.47 |  |  |  |  |
| 1. Model for maladaptive coping | | | | | | | | | | | | | | | |
|  | β | *b* | *SE* | *t* | 95% CI | β | *b* | *SE* | *t* | 95% CI | β | *b* | *SE* | *t* | 95% CI |
| Empathy | -.10 | -.12 | .03 | -3.64*** | -.19, -.06 | -.11 | -.12 | .03 | -3.75*** | -.19, -.06 | -.11 | -.12 | .03 | -3.76*** | -.19, -.06 |
| SES |  |  |  |  |  | -.08 | -.05 | .02 | -2.57* | -.08, -.01 | -.08 | -.05 | .02 | -2.58* | -.08, -.01 |
| Empathy× SES |  |  |  |  |  |  |  |  |  |  | -.01 | -.01 | .02 | -.41 | -.05, .03 |
| *R*^2^ | .03 |  |  |  |  | .05 |  |  |  |  | .05 |  |  |  |  |
| Adjusted *R*^2^ | .03 |  |  |  |  | .04 |  |  |  |  | .04 |  |  |  |  |
| *F* | 13.22 |  |  |  |  | 10.01 |  |  |  |  | 6.71 |  |  |  |  |

* *p* < .05, *** *p* < .001

Similar to Study 1, we conducted simple slope analyses to assess the effect of empathy on adaptive coping at different levels of SES (i.e., +/- 1 *SD* of the mean). Results suggest that empathy has a positive relationship for both lower SES (-1 SD than mean) and higher SES (+1 SD than mean) individuals, but the influence was bigger for the relatively poor, *b* = .31, *SE* = .04, *t*(391) = 8.50, *p* < .001, 95% *CI* [.24, .38], than the relatively rich, *b* = .20, *SE* = .04, *t*(391) = 5.10, *p* < .001, 95% *CI* [.12,.27]. This finding supports the poor-protection hypothesis, helping the poor to use more adaptive coping.

Figure S2 displays the interaction results between empathy and social support, Figure S3 illustrates the interaction results between empathy and maladaptive coping.

**S2 Fig. Relationship between empathy and social support in Study 2.**

1. Simple slope result for +/- 1SD SES individuals on social support. (B) Estimated coefficient of empathy on social support for different SES individuals.

**S3 Fig. Relationship between empathy and maladaptive coping in Study 2.**

1. Simple slope result for +/- 1SD SES individuals on maladaptive coping. (B) Estimated coefficient of empathy on maladaptive coping for different SES individuals.
