# Supplementary material for: Caring helps: Trait empathy is related to better coping strategies and differs in the poor versus the rich
Source: PLoS One. 2019 Mar 27;14(3):e0213142. doi: 10.1371/journal.pone.0213142 (PMC6436718; doi:10.1371/journal.pone.0213142)
Supplement: S3 File — (DOCX) [file pone.0213142.s003.docx]

**Study 3**

**Method**

**Participants**

We recruited 2000 participants from the United States via the survey panel Tellwut. After removing participants who had duplicated entries and those who had incomplete cases, 1477 participants (Male = 307, *M*_age_ (*SD*) = 22.48 (13.15)) entered the final analysis. All procedures and measures were identical to Study 1 and Study 2.

**Results**

Similar to Studies 1 and 2, in Study 3, participants’ perspective taking (PT) scores ranged from 0 to 4, with mean score 2.68 and SD .68. PT was positively related to adaptive coping (*b* = .23, *SE* = .01, *t*(1411) = 20.64, *p* < .001, 95%*CI* [.21, .25]) and use of social support (*b* = .16, *SE* = .02, *t*(1411) = 8.37, *p* < .001, 95%*CI* [.12, .19]), and was negatively related to maladaptive coping (*b* = -.15, *SE* = .02, *t*(1411) = -8.77 *p* < .001, 95%*CI* [-.18, -.11]). When considering PT and SES together, the same direction preserved (*ps* < .001). However, there was no interaction between PT and SES on any coping strategies (*ps* > .1).

**Table A. Study 3 Participants’ Means and Standard Deviations on the Measure of SES, Empathy, and Coping Strategies**

|  | SES | Empathy | Adaptive coping | Social support | Maladaptive coping |
| --- | --- | --- | --- | --- | --- |
| *M* | 5.04 | 2.93 | 2.46 | 2.21 | 1.65 |
| *SD* | 1.96 | .71 | .33 | .49 | .44 |

As in Studies 1-2, we found that empathy was positively related to adaptive coping, social support, and negatively related to maladaptive coping. SES was also positively linked to adaptive coping and social support; however, in contrast to our previous studies, we found a positive, rather than negative, link between SES and maladaptive coping. Yet the empathy X SES interaction was significant for both social support and maladaptive coping. Detailed results were displayed in Table B.

**Table B. Hierarchical Regression Models Predicting Coping Strategies in Study 3**

| 1. Model for adaptive coping | | | | | | | | | | | | | | | |
| --- | --- | --- | --- | --- | --- | --- | --- | --- | --- | --- | --- | --- | --- | --- | --- |
|  | β | *b* | *SE* | *t* | 95% CI | β | *b* | *SE* | *t* | 95% CI | β | *b* | *SE* | *t* | 95% CI |
| Empathy | .11 | .16 | .01 | 13.89*** | .14, .18 | .12 | .17 | .01 | 14.64*** | .15, .19 | .12 | .17 | .01 | 15.57*** | .15, .19 |
| SES |  |  |  |  |  | .06 | .03 | .00 | 6.87*** | .02, .04 | .06 | .03 | .00 | 6.80*** | .02, .04 |
| Empathy× SES |  |  |  |  |  |  |  |  |  |  | .00 | .00 | .01 | -.23 | -.01, .01 |
| *R*^2^ | .12 |  |  |  |  | .15 |  |  |  |  | .15 |  |  |  |  |
| Adjusted *R*^2^ | .12 |  |  |  |  | .15 |  |  |  |  | .15 |  |  |  |  |
| *F* | 192.90 |  |  |  |  | 123.20 |  |  |  |  | 82.07 |  |  |  |  |
| 1. Model for social support | | | | | | | | | | | | | | | |
|  | β | *b* | *SE* | *t* | 95% CI | β | *b* | *SE* | *t* | 95% CI | β | *b* | *SE* | *t* | 95% CI |
| Empathy | .10 | .15 | .02 | 8.21*** | .11, .18 | .11 | .16 | .02 | 8.98*** | .12, .19 | .11 | .16 | .02 | 8.95*** | .13,.19 |
| SES |  |  |  |  |  | .09 | .05 | .01 | 7.55*** | .04,.06 | .09 | .05 | .01 | 7.32*** | .03,.06 |
| Empathy× SES |  |  |  |  |  |  |  |  |  |  | -.03 | -.02 | .01 | -2.74** | -.04, -.01 |
| *R*^2^ | .05 |  |  |  |  | .08 |  |  |  |  | .09 |  |  |  |  |
| Adjusted *R*^2^ | .04 |  |  |  |  | .08 |  |  |  |  | .09 |  |  |  |  |
| *F* | 67.42*** |  |  |  |  | 63.52*** |  |  |  |  | 45.05*** |  |  |  |  |
| 1. Model for maladaptive coping | | | | | | | | | | | | | | | |
|  | β | *b* | *SE* | *t* | 95% CI | β | *b* | *SE* | *t* | 95% CI | β | *b* | *SE* | *t* | 95% CI |
| Empathy | -.11 | -.16 | .02 | -10.04*** | -.19, -.13 | -.11 | -.16 | .02 | -9.73*** | -.19, -.13 | -.11 | -.16 | .02 | -9.96*** | -.19, -.13 |
| SES |  |  |  |  |  | .04 | .02 | .01 | 3.81*** | .01, .03 | .04 | .02 | .01 | 3.37** | .01, .03 |
| Empathy× SES |  |  |  |  |  |  |  |  |  |  | -.06 | -.05 | .01 | -5.91*** | -.06, -.03 |
| *R*^2^ | .07 |  |  |  |  | .08 |  |  |  |  | .10 |  |  |  |  |
| Adjusted *R*^2^ | .07 |  |  |  |  | .07 |  |  |  |  | .10 |  |  |  |  |
| *F* | 100.90*** |  |  |  |  | 58.19*** |  |  |  |  | 51.38*** |  |  |  |  |

** *p* < .01, *** *p* < .001

In the last step, we explored the effect of empathy on social support and maladaptive coping for high (1SD above mean) and low (1SD below mean) SES individuals. Results suggest that the positive influence of empathy on social support was stronger for lower SES, *b* = .20, *SE* = .02, *t*(1413) = 8.46, *p* < .001, 95%*CI* [.16, .25], than higher SES people, *b* = .11, *SE* = .02, *t*(1413) = 4.55, *p* < .001, 95%*CI* [.06, .16]. Empathy was also negatively related to the usage of maladaptive coping, and this effect was stronger for the rich, *b* = -.25, *SE* = .02, *t*(1413) = -11.19, *p* < .001, 95%*CI* [-.29, -.20], than the poor, *b* = -.07, *SE* = .02, *t*(1413) = -3.18, *p* < .01, 95%*CI* [-.11, -.03]. Thus, for social support, we found support for the poor-protection hypothesis, yet for maladaptive coping, we found support for the rich-protection hypothesis.

**S4 Fig. Relationship between empathy and adaptive coping in Study 3.**

1. Simple slope result for +/- 1SD SES individuals on adaptive coping. (B) Estimated coefficient of empathy on adaptive coping for different SES individuals.

**S5 Fig. Relationship between empathy and social support in Study 3.**

1. Simple slope result for +/- 1SD SES individuals on social support. (B) Estimated coefficient of empathy on social support for different SES individuals.

**S6 Fig. Relationship between empathy and maladaptive coping in Study 3.**

1. Simple slope result for +/- 1SD SES individuals on maladaptive coping. (B) Estimated coefficient of empathy on maladaptive coping for different SES individuals.

Study 3 partially replicated the results in Study 1 and 2. First, empathy’s positive main effect on adaptive coping and social support fully replicated the results in Study 1 and 2. For the empathy x SES interaction, there was a negative interaction of social support, which was different from Study 1 (positive) and 2 (no significance). There was a positive interaction on adaptive coping and a negative interaction on maladaptive coping, both replicating Study 1 and Study 2. Since Study 3 could not solve the inconsistency problem for the interaction on social support, we decided to change to another survey panel and replicate the same study with another big sample.
