# Supplementary material for: Caring helps: Trait empathy is related to better coping strategies and differs in the poor versus the rich
Source: PLoS One. 2019 Mar 27;14(3):e0213142. doi: 10.1371/journal.pone.0213142 (PMC6436718; doi:10.1371/journal.pone.0213142)
Supplement: S4 File — (DOCX) [file pone.0213142.s004.docx]

**Study 4**

**Method**

**Participants**

We recruited 1200 participants from the United States via the survey panel Cint. After removing participants who had duplicated entries and those who had incomplete cases, 1132 participants (Male = 537, *M*_age_ (*SD*) = 41.06 (12.55)) entered the final analysis. Measures and procedures were identical to the previous studies.

**Results**

Participants’ perspective taking (PT) scores ranged from 0 to 4, with mean score 2.58 and SD .61. PT was positively related to adaptive coping (*b* = .35, *SE* = .03, *t*(1058) = 11.41, *p* < .001, 95%*CI* [.29, .42]) and use of social support (*b* = .28, *SE* = .04, *t*(1058) = 6.79, *p* < .001, 95%*CI* [.20, .35]), and was negatively related to maladaptive coping (*b* = -.12, *SE* = .04, *t*(1058) = -3.44, *p* < .001, 95%*CI* [-.19, -.05]). When considering PT and SES together, the same direction preserved (*ps* < .001). However, there was no interaction between PT and SES on any coping strategies (*ps* > .1).

**Table A. Study 4 Participants’ Means and Standard Deviations on the Measure of SES, Empathy, and Coping Strategies**

|  | SES | Empathy | Adaptive coping | Social support | Maladaptive coping |
| --- | --- | --- | --- | --- | --- |
| *M* | 5.72 | 2.72 | 2.86 | 2.45 | 1.95 |
| *SD* | 2.15 | .67 | .69 | .86 | .73 |

Statistical analysis procedure was the same as in Study 1-3. As before, we centered SES and empathy, and used the centered score in the regression models. In this sample, we found that (1) empathy was positively related to adaptive coping and social support, and negatively related to maladaptive coping. This result fully replicates those in Study 1-3. (2) When considering empathy and SES at the same time, SES was positively related to adaptive coping and social Support, which was the same trend for Study 1-3, but also positively related to maladaptive coping, which was same as in Study 3, but opposite to Study 1-2. (3) When considering SES as a moderator for empathy and coping, we found that it moderated the relationship for all three coping styles. More specifically, there was a negative moderation between SES and empathy for adaptive coping, social support, and maladaptive coping. Detailed results were shown in Table B below.

**Table B. Hierarchical Regression Models Predicting Coping Strategies in Study 4**

| 1. Model for adaptive coping | | | | | | | | | | | | | | | |
| --- | --- | --- | --- | --- | --- | --- | --- | --- | --- | --- | --- | --- | --- | --- | --- |
|  | β | *b* | *SE* | *t* | 95% CI | β | *b* | *SE* | *t* | 95% CI | β | *b* | *SE* | *t* | 95% CI |
| Empathy | .14 | .20 | .03 | 6.62*** | .14, .26 | .16 | .23 | .03 | 7.82 | .17, .29 | .15 | .22 | .03 | 7.38*** | .16, .28 |
| SES |  |  |  |  |  | .16 | .07 | .01 | 7.73*** | .05,.09 | .15 | 0.07 | 0.01 | 7.17*** | .05, .09 |
| Empathy× SES |  |  |  |  |  |  |  |  |  |  | -.08 | -.05 | .01 | -3.85*** | -.08, -.03 |
| *R*^2^ | .04 |  |  |  |  | .09 |  |  |  |  | 0.10 |  |  |  |  |
| Adjusted *R*^2^ | .04 |  |  |  |  | .09 |  |  |  |  | 0.10 |  |  |  |  |
| *F* | 43.80*** |  |  |  |  | 53.04*** |  |  |  |  | 40.77*** |  |  |  |  |
| 1. Model for social support | | | | | | | | | | | | | | | |
|  | β | *b* | *SE* | *t* | 95% CI | β | *b* | *SE* | *t* | 95% CI | β | *b* | *SE* | *t* | 95% CI |
| Empathy | .09 | .13 | .04 | 3.27** | .05, .20 | .12 | .18 | .04 | 4.77*** | .10, .25 | .11 | .16 | .04 | 4.29*** | .09,.23 |
| SES |  |  |  |  |  | .26 | .12 | .01 | 10.18*** | .10, .14 | .24 | .11 | .01 | 9.56*** | .09,.14 |
| Empathy× SES |  |  |  |  |  |  |  |  |  |  | -.11 | -.08 | .02 | -4.36*** | -.11,- .04 |
| *R*^2^ | .01 |  |  |  |  | .10 |  |  |  |  | .11 |  |  |  |  |
| Adjusted *R*^2^ | .01 |  |  |  |  | .10 |  |  |  |  | .11 |  |  |  |  |
| *F* | 10.71* |  |  |  |  | 57.5*** |  |  |  |  | 45.32*** |  |  |  |  |
| 1. Model for maladaptive coping | | | | | | | | | | | | | | | |
|  | β | *b* | *SE* | *t* | 95% CI | β | *b* | *SE* | *t* | 95% CI | β | *b* | *SE* | *t* | 95% CI |
| Empathy | -.14 | -.20 | .03 | -6.14*** | -.26, -.14 | -.11 | -.17 | .03 | -5.22*** | -.23, -.11 | -.14 | -.19 | .03 | -6.30*** | -.26, -.14 |
| SES |  |  |  |  |  | .16 | .08 | .01 | 7.41*** | .06, .10 | .14 | .06 | .01 | 6.41*** | .04,.08 |
| Empathy× SES |  |  |  |  |  |  |  |  |  |  | -.18 | -.12 | .02 | -8.40*** | -.15, -.10 |
| *R*^2^ | 0.03 |  |  |  |  | .08 |  |  |  |  | .14 |  |  |  |  |
| Adjusted *R*^2^ | 0.03 |  |  |  |  | .08 |  |  |  |  | .14 |  |  |  |  |
| *F* | 37.70*** |  |  |  |  | 47.41*** |  |  |  |  | 57.21*** |  |  |  |  |

* *p* < .05, ** *p* < .01, *** *p* < .001

Similar to previous studies, we conducted simple slope analyses for individuals with relatively low (1SD below mean) and relatively high (1SD above mean) SES. Empathy was more strongly positively related to adaptive coping for the poor, *b* = .33, *SE* = .04, *t*(1059) = 8.41, *p* < .001, 95%CI [.26, .41], than the rich, *b* = .10, *SE* = .05, *t*(1059) = 2.29, *p* =.02, 95%CI [.01, .19]. Empathy also was positively related to using more social support for people with low SES, *b* = .32, *SE* = .05, *t*(1059) = 6.50, *p* < .001, 95%CI [.23, .42], but not high SES, *b* = -.01, *SE* = .06, *t*(1059) = -.11, *p* > .5, 95%CI [-.12, .10]. For maladaptive coping, empathy was negatively related to the usage of maladaptive coping for the rich, *b* = -.47, *SE* = .05, *t*(1059) = -9.87, *p* < .001, 95%CI [-.56, -.37], but not for the poor., *b* = .07, *SE* = .04, *t*(1059) = 1.69, *p* = .09, 95%CI [-.01, .15]. These results again lend support to both of our competing hypotheses: in support of the poor-protection hypothesis, empathy increases the usage of adaptive coping and social support for the poor; and in support of the rich-protection hypothesis, empathy decreases the usage of maladaptive coping for the rich.

Similar to Studies 1-3, we display the interaction results in two ways, shown in S7-S9 Fig.

**S7 Fig. Relationship between empathy and adaptive coping in Study 4.**

1. Simple slope result for +/- 1SD SES individuals on adaptive coping. (B) Estimated coefficient of empathy on adaptive coping for different SES individuals.

**S8 Fig. Relationship between empathy and social support in Study 4.**

1. Simple slope result for +/- 1SD SES individuals on social support. (B) Estimated coefficient of empathy on social support for different SES individuals.

**S9 Fig. Relationship between empathy and maladaptive coping in Study 4.**

1. Simple slope result for +/- 1SD SES individuals on maladaptive coping. (B) Estimated coefficient of empathy on maladaptive coping for different SES individuals.
