# Supplementary material for: Caring helps: Trait empathy is related to better coping strategies and differs in the poor versus the rich
Source: PLoS One. 2019 Mar 27;14(3):e0213142. doi: 10.1371/journal.pone.0213142 (PMC6436718; doi:10.1371/journal.pone.0213142)
Supplement: S5 File — (DOCX) [file pone.0213142.s005.docx]

**Study 5**

**Table A. Hierarchical Regression Results for Social Support**

|  | Step 1 | | | | Step 2 | | | | Difference between Step 1 and Step 2 |  |
| --- | --- | --- | --- | --- | --- | --- | --- | --- | --- | --- |
|  | b | SE | t | 95% CI | b | SE | t | 95% CI |  | |
| SES | -.02 | .03 | -.63 | -.07, .04 | .03 | .05 | .70 | -.06, .13 |  | |
| GG | .23 | .10 | 2.33* | .04, .42 | .22 | .10 | 2.22* | .03, .41 |  | |
| AG | .05 | .10 | .52 | -.14, .24 | .05 | .10 | .50 | -.14, .24 |  | |
| SES x GG |  |  |  |  | -.07 | .07 | -.93 | -.20, .07 |  | |
| SES x AG |  |  |  |  | -.08 | .07 | -1.25 | -.22, .05 |  | |
| R^2^ | .02 |  |  |  | .02 |  |  |  |  | |
| Adjusted R^2^ | .01 |  |  |  | .01 |  |  |  |  | |
| F | 2.12 |  |  |  | 1.61 |  |  |  | .433 | |

- *p* < .05

**S10 Fig. Interaction between Genotype and SES on Social Support in Study 5.**

1. Line plot. (B) Bar plot with standard deviation.
