# Supplementary material for: Caring helps: Trait empathy is related to better coping strategies and differs in the poor versus the rich
Source: PLoS One. 2019 Mar 27;14(3):e0213142. doi: 10.1371/journal.pone.0213142 (PMC6436718; doi:10.1371/journal.pone.0213142)
Supplement: S6 File — (DOCX) [file pone.0213142.s006.docx]

**Correlation between subjective SES and Positive Reappraisal in Studies 1-5**

Study 1: *r* = .19, *t*(337) = 3.47, *p* < .001

Study 2: *r* = .17, *t*(395) = 3.50, *p* < .001

Study 3: *r* = .14, *t*(1475) = 5.44, *p* < .001

Study 4: *r* = .16, *t*(1127) = 5.51, *p* < .001

Study 5: *r* = .06, *t*(353) = 1.05, *p* = .30

**Correlation between subjective SES and Empathic Concern in Studies 1-5**

Study 1: *r* = -.04, *t*(337) = -.78, *p* = .44

Study 2: *r* = -.04, *t*(395) = -.79, *p* = .43

Study 3: *r* = -.08, *t*(1415) = -3.20, *p* = .001

Study 4: *r* = -.14, *t*(1061) = -4.59, *p* < .001

Study 5: *r* = -.12, *t*(353) = -2.31, *p* = .02
