# Supplementary figures and images for: Caring helps: Trait empathy is related to better coping strategies and differs in the poor versus the rich
Source: PLoS One. 2019 Mar 27;14(3):e0213142. doi: 10.1371/journal.pone.0213142 (PMC6436718; doi:10.1371/journal.pone.0213142)

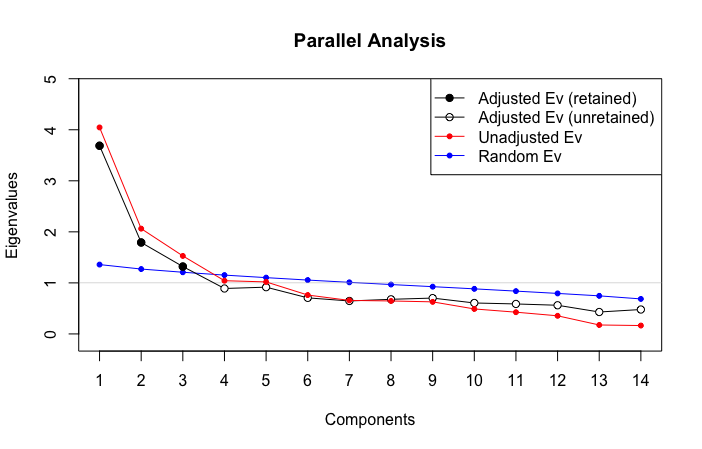

Supplement: S1 Fig — (PNG) [file pone.0213142.s007.png]

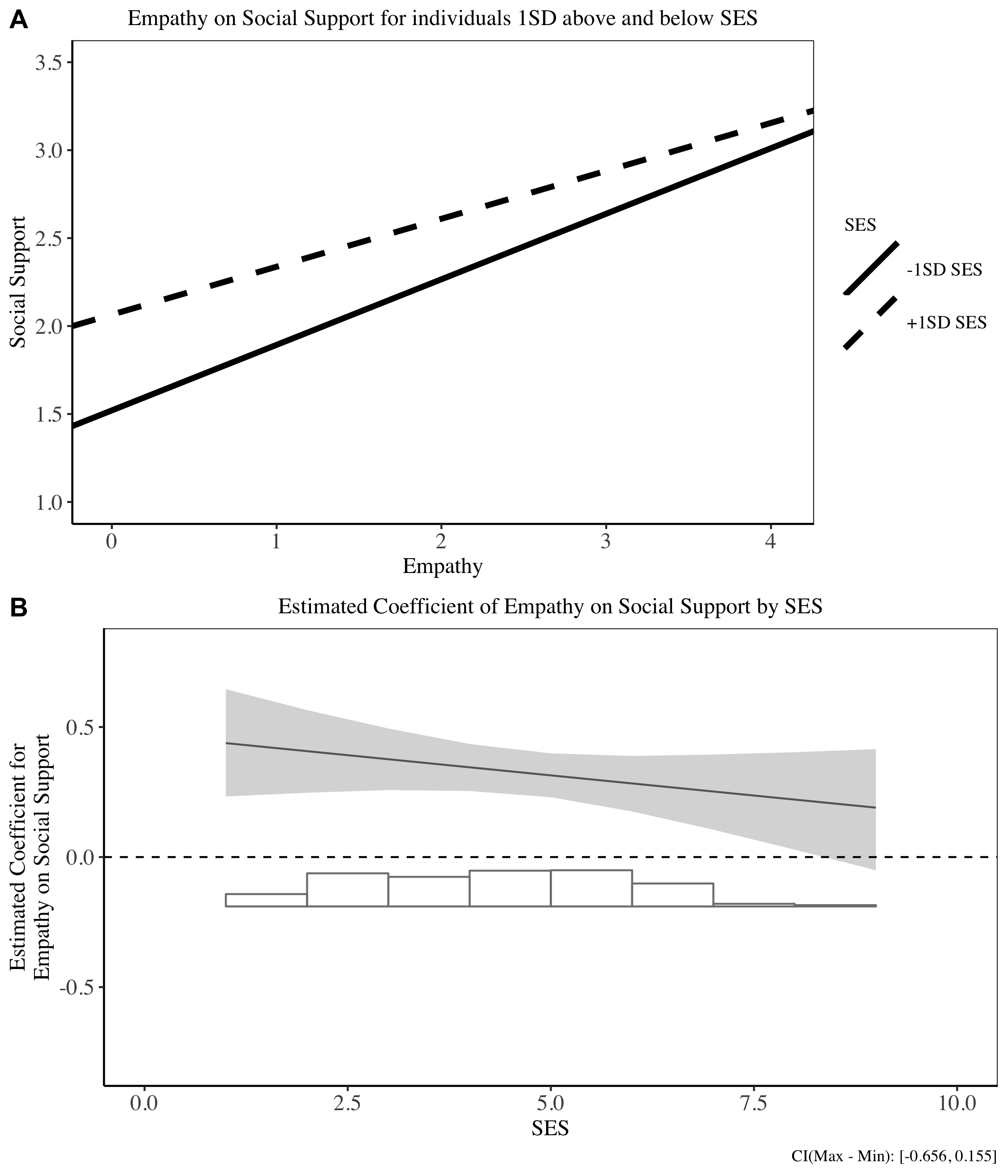

Supplement: S2 Fig — (TIFF) [file pone.0213142.s008.tiff]

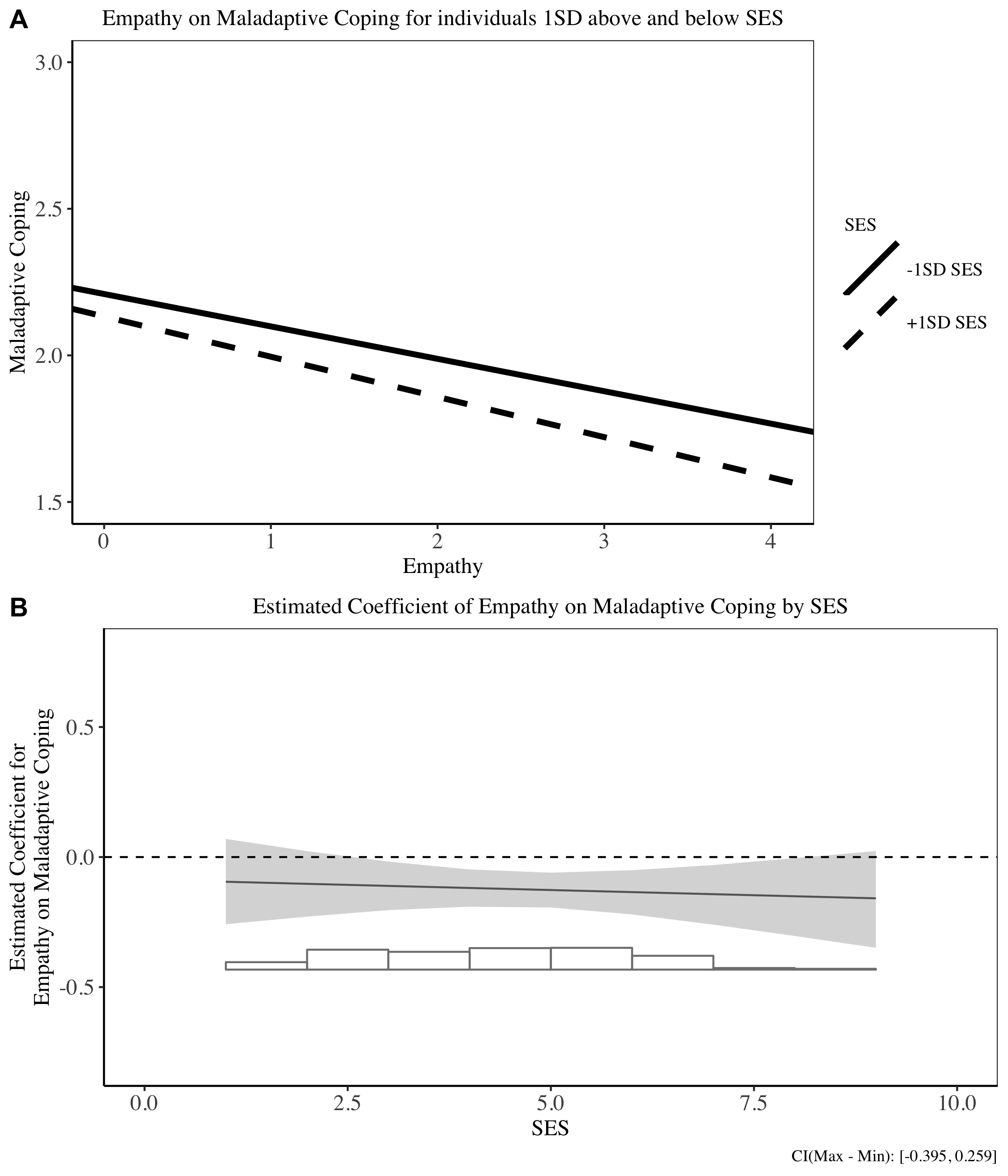

Supplement: S3 Fig — (TIFF) [file pone.0213142.s009.tiff]

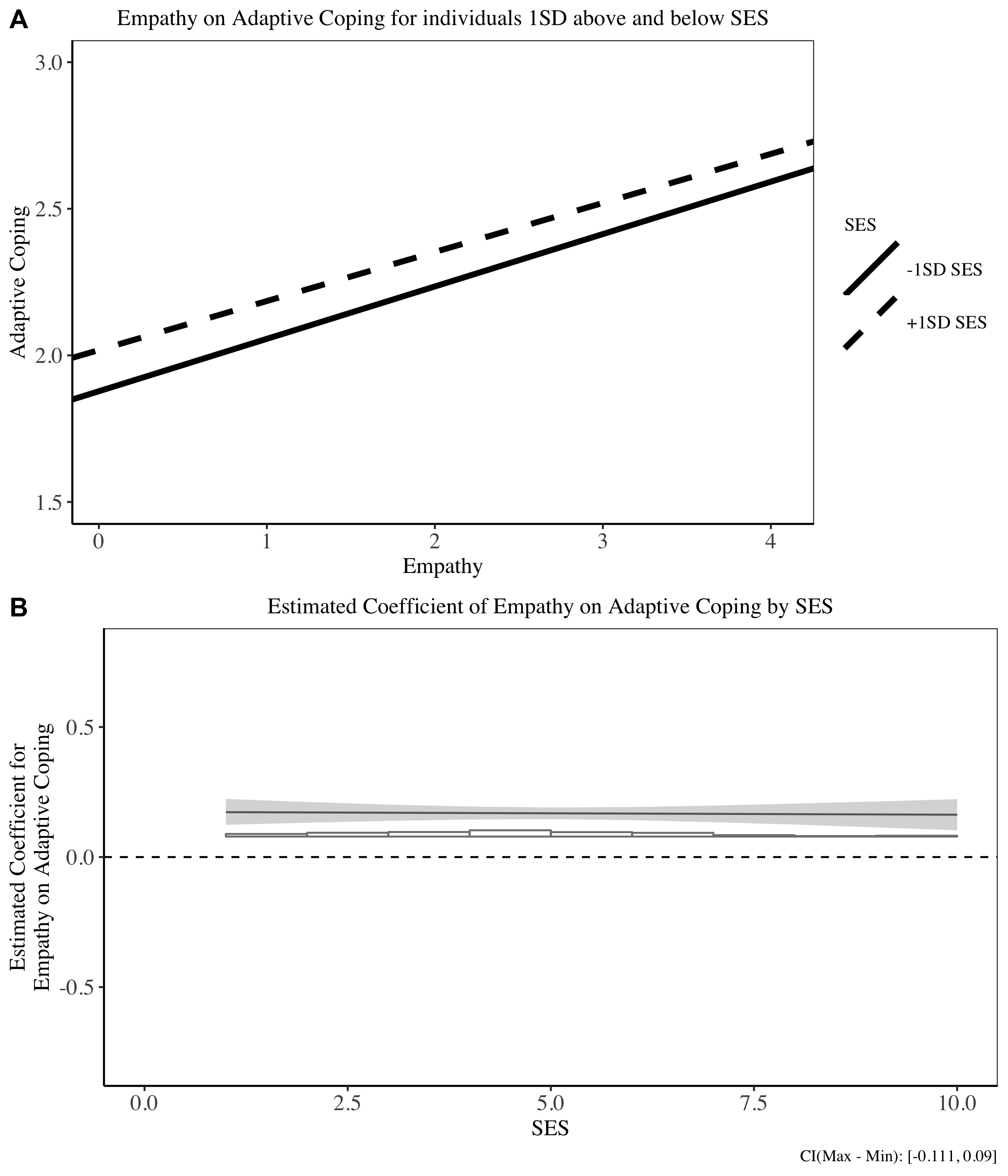

Supplement: S4 Fig — (TIFF) [file pone.0213142.s010.tiff]

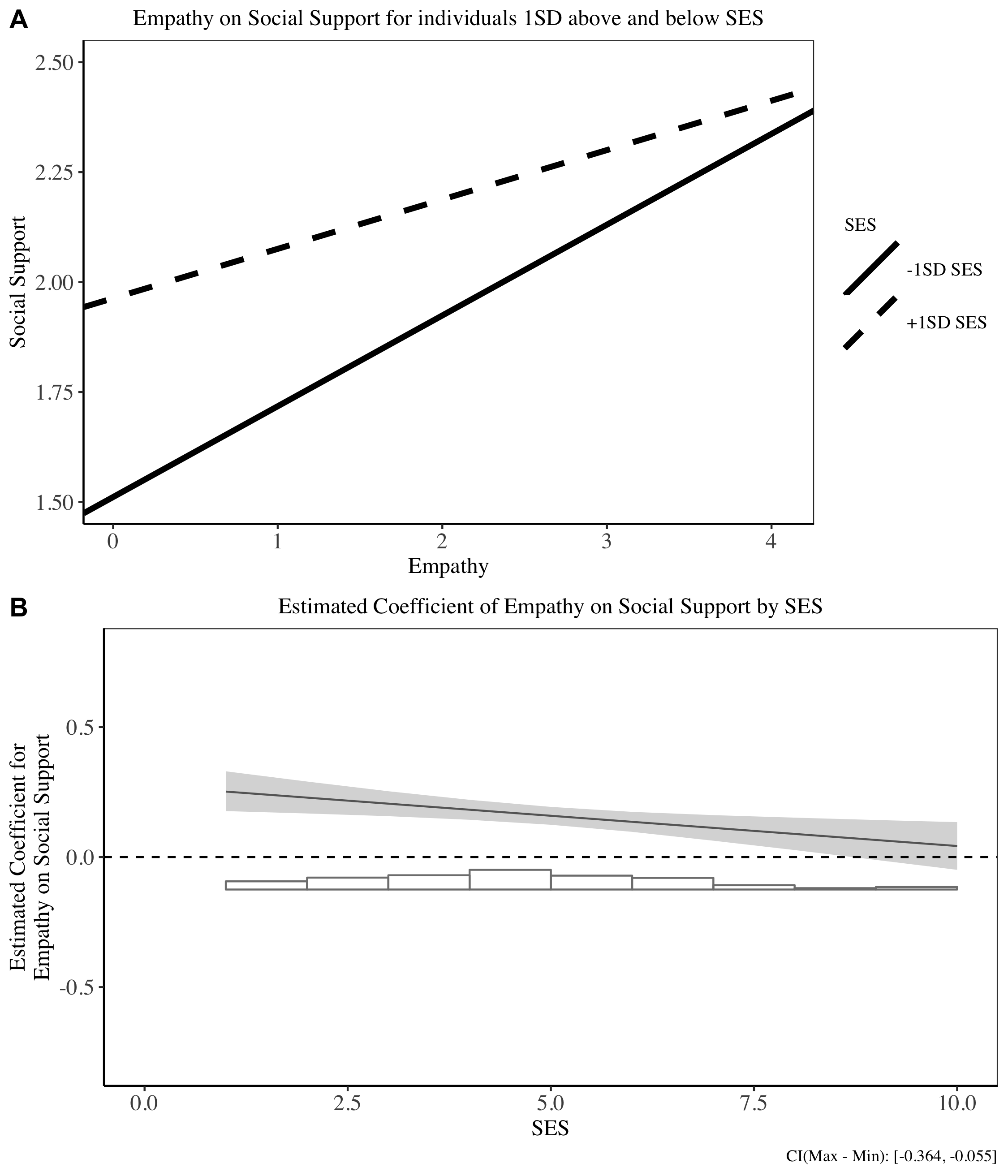

Supplement: S5 Fig — (TIFF) [file pone.0213142.s011.tiff]

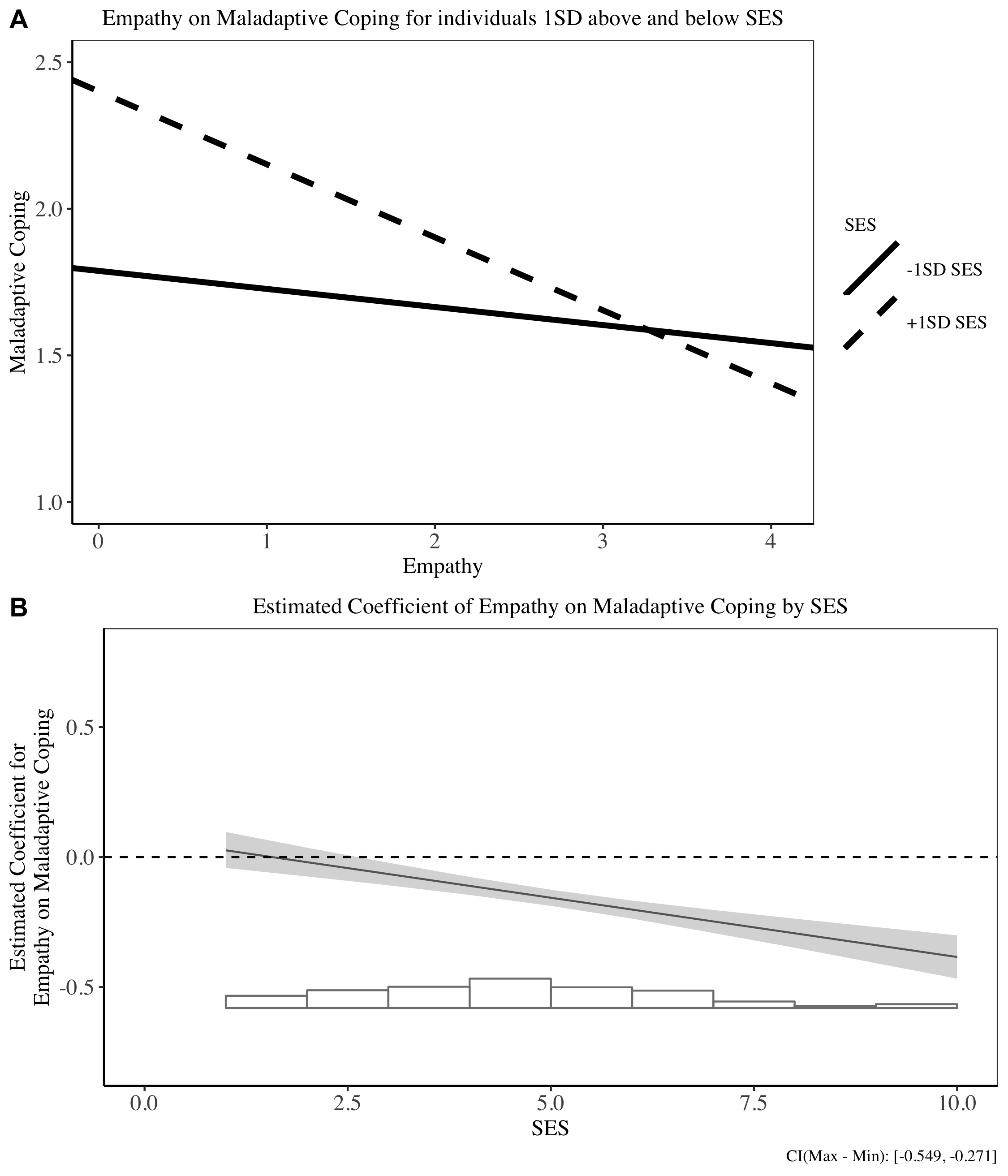

Supplement: S6 Fig — (TIFF) [file pone.0213142.s012.tiff]

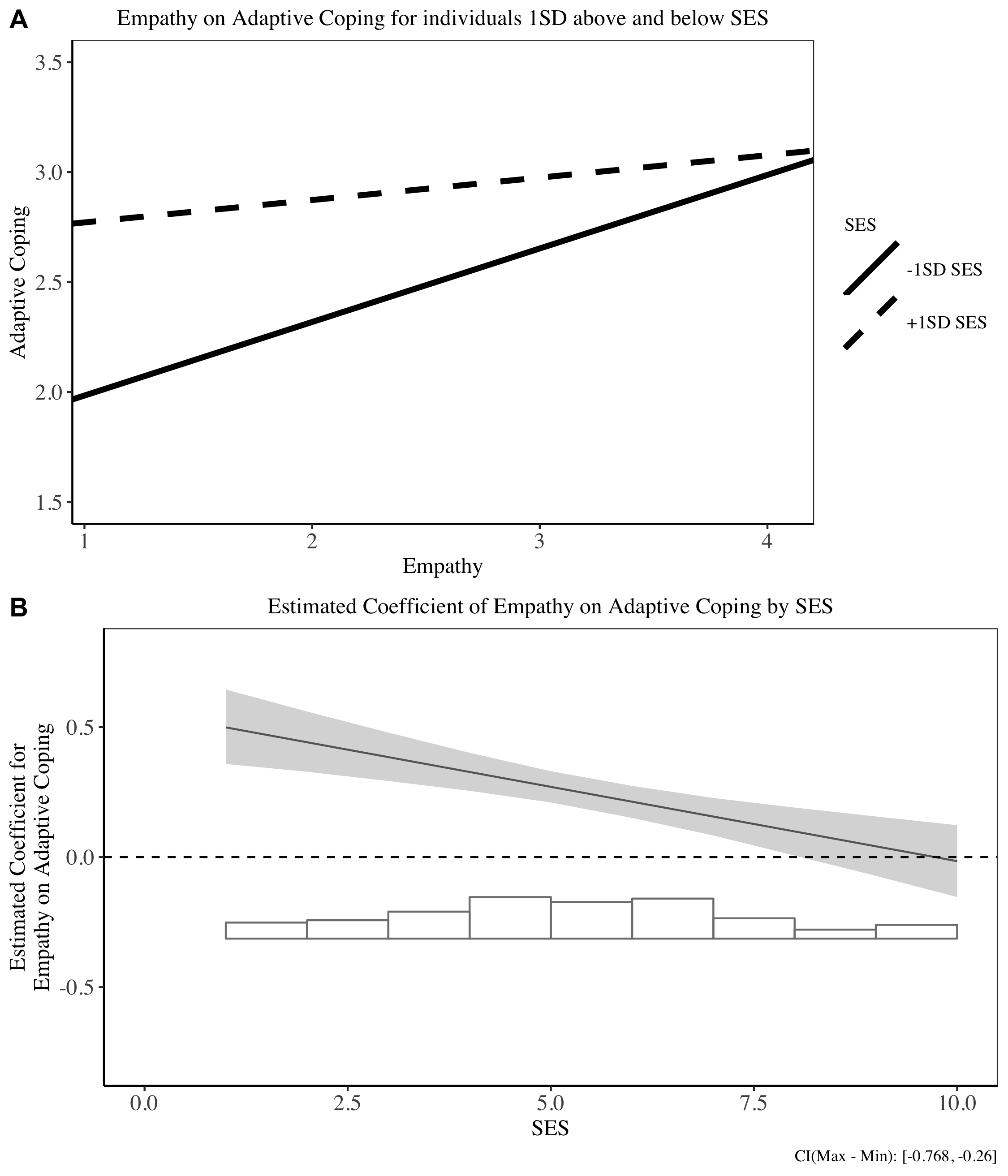

Supplement: S7 Fig — (TIFF) [file pone.0213142.s013.tiff]

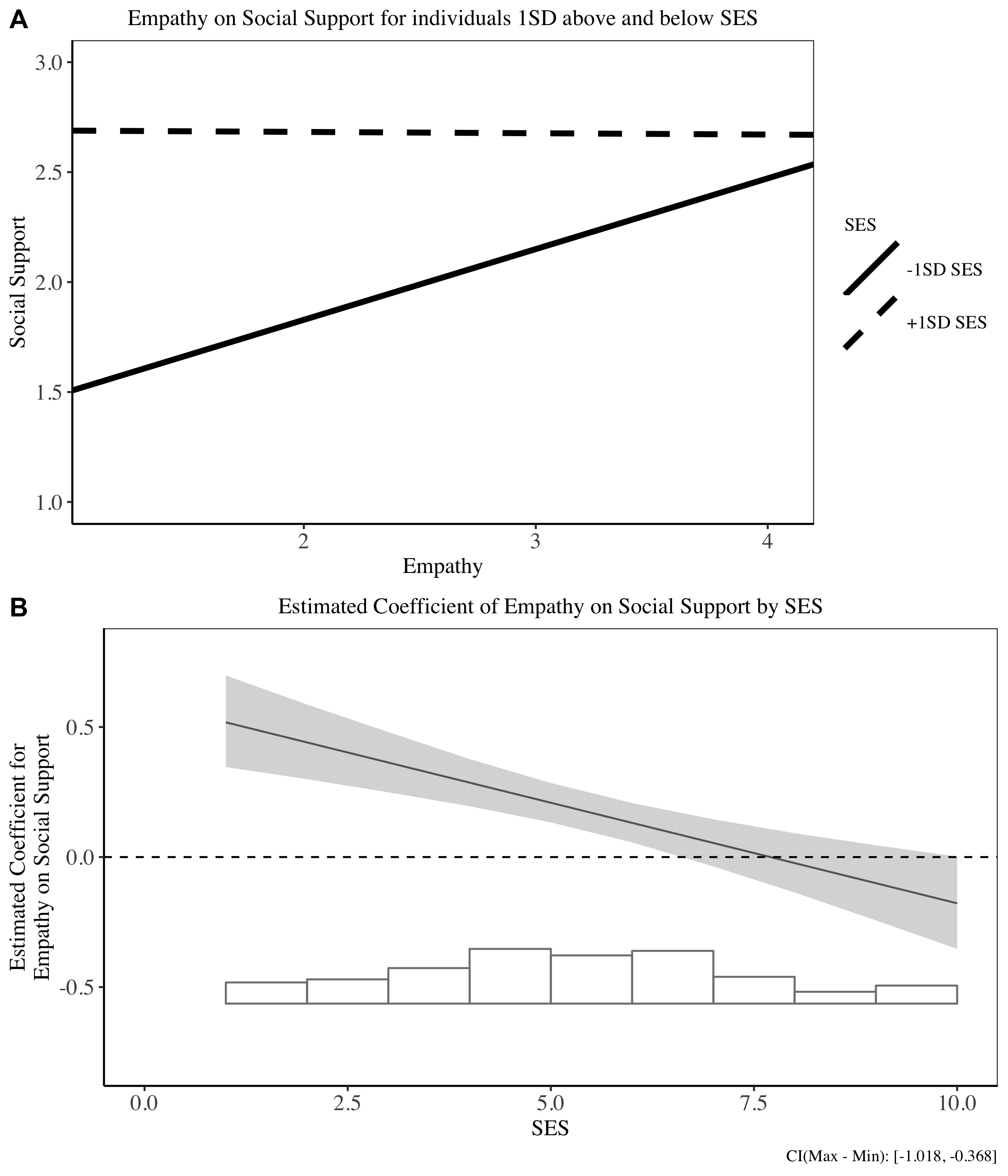

Supplement: S8 Fig — (TIFF) [file pone.0213142.s014.tiff]

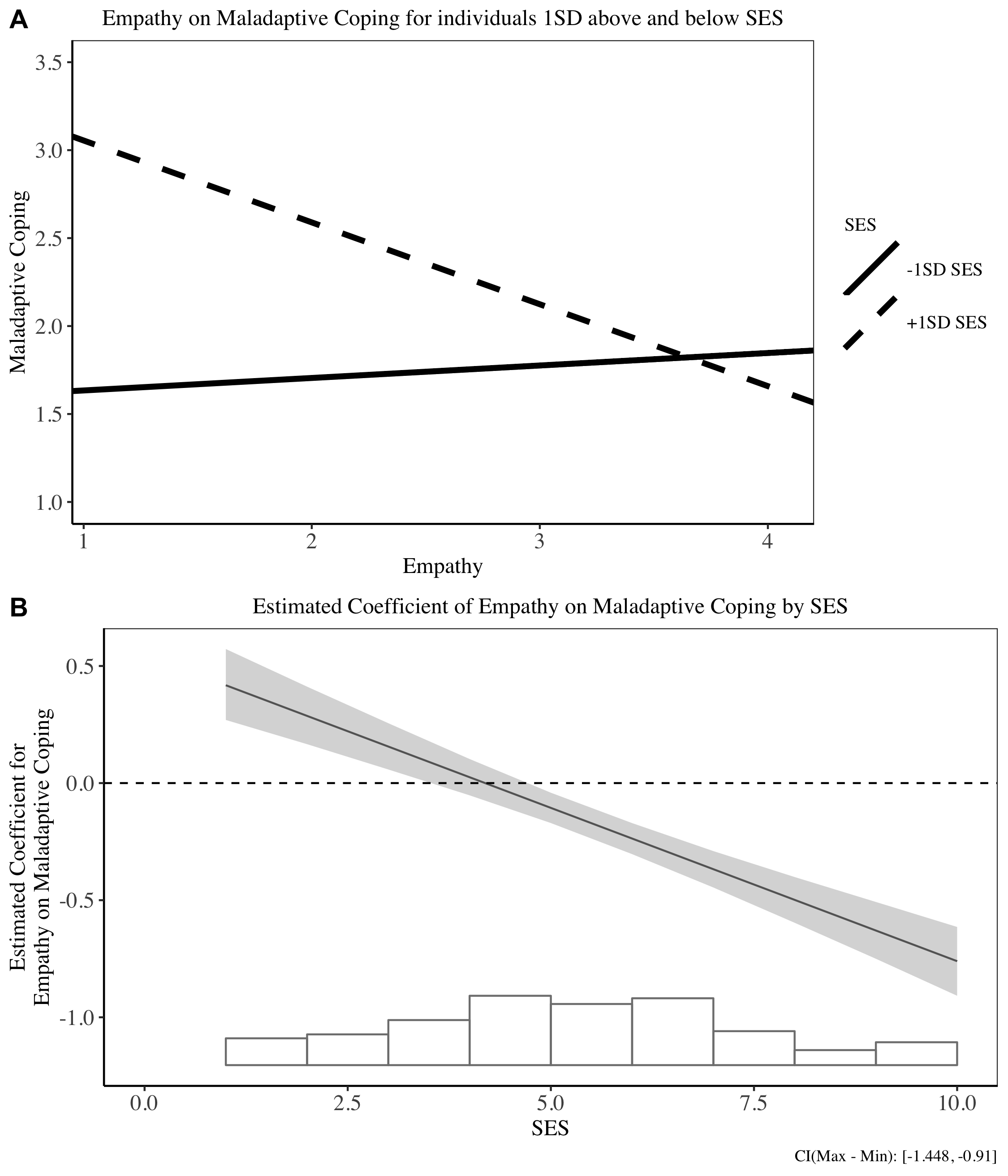

Supplement: S9 Fig — (TIFF) [file pone.0213142.s015.tiff]

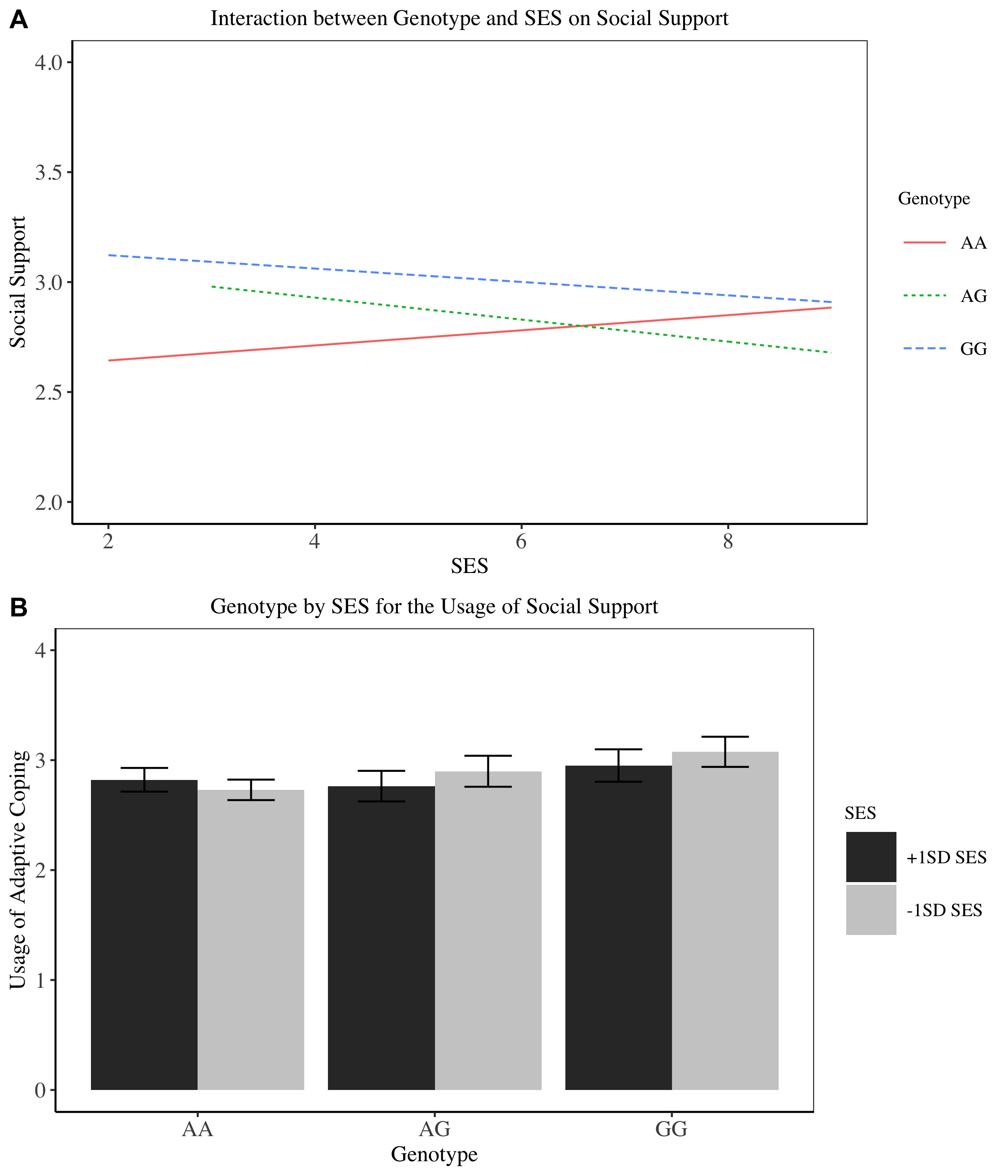

Supplement: S10 Fig — (TIFF) [file pone.0213142.s016.tiff]
